# Adult Striatal Neurogenesis—A Comparative Approach Between Pigeons, Mice, Macaques, and Human

**DOI:** 10.1002/cne.70107

**Published:** 2025-11-02

**Authors:** Christina Herold, Erhan Karsli, Nicole Delhaes, Julia Mehlhorn, Hans Bidmon, Katrin Amunts

**Affiliations:** ^1^ Cécile and Oskar Vogt‐Institute for Brain Research, Medical Faculty University Hospital and Heinrich Heine University Düsseldorf Düsseldorf Germany; ^2^ Institute for Anatomy I, Medical Faculty University Hospital Düsseldorf and Heinrich Heine University Düsseldorf Düsseldorf Germany; ^3^ Institute of Neuroscience and Medicine INM‐1 Research Center Jülich Jülich Germany

**Keywords:** adult neurogenesis, avian, basal ganglia, birds, BrdU, caudoputamen, DCX, globus pallidus, nucleus accumbens, mammals, primates, rodents, striatum

## Abstract

Adult neurogenesis describes the formation of new neurons in the adult brain, a process that is fundamental to related functions, particularly in the hippocampus. Although studies reported adult striatal neurogenesis in humans, the phenomenon is still understudied in those regions. Thus, to gain a deeper understanding in different species, the expression of neurogenic markers was quantitatively analyzed in striatal subregions of pigeons and mice. Further, in macaques and human a detailed analysis of the subventricular zone (SVZ) was performed and the human caudate nucleus was qualitatively examined. The results show higher neuronal plasticity in striatal subregions of pigeons compared to mice, as reflected by higher numbers of Bromodeoxyuridine (BrdU)+, BrdU+/Doublecortin+, Doublecortin+, and BrdU+/Neuronal nuclei marker+ cells. Analysis of BrdU+/glial fibrillary acidic protein (GFAP)+ signals indicated further higher gliogenesis/potential stem cell division in pigeons. As newborn striatal neurons may arise from stem cell niches in the SVZ, active proliferation was analyzed with (sex determining region Y)‐box 2, GFAP, and Ki‐67 in macaques and humans. Specific subdivisions of the SVZ were identified, with GFAP and Ki‐67 differentially distributed. Additionally, signs of persistent neuronal plasticity were observed with Doublecortin+ cells in the human caudate nucleus but not in the macaque. The higher levels of striatal adult neurogenesis in pigeons and perspectives of useful methods may encourage the use of birds to investigate the functional role of this phenomenon and may facilitate our understanding of neuronal plasticity even in the human striatum in the future.

## Graphical abstract

Striatal adult neurogenesis is an understudied field. Here, we add new quantitative data showing that pigeons provide high levels of adult neurogenesis in the striatum compared to mice. Besides, we show that macaque and human differ in their potential to produce adult‐born neurons at least in the caudate nucleus.

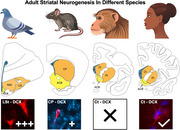

**Abbreviations**
ACB: nucleus accumbens
avV‐SVZ: anteroventral ventricular‐ subventricular zone
BrdU: Bromodeoxyuridine
CP: caudoputamen
CPi: intermediate caudoputamen
CR: calretinin
CY3: Cyanine 3
DAPI: 4′,6‐diamidin‐2‐phenylindole
DCX: Doublecortin
Dlx‐2: Distal‐Less Homeobox 2
EGFR: epidermal growth factor receptor
FS: fundus striatum
GABA: gamma aminobutyric acid
GFAP: glial fibrillary acidic protein
GP: globus pallidus
HVC: higher vocal center
ISt: intermediate striatum (former INP, nucleus intrapeduncularis)
Ki67: antigen KI‐67
LSt: lateral striatum
MSt: medial striatum
NeuN: Neuronal nuclei marker
PARV: parvalbumin
PCNA: proliferating cell nuclear antigen
RMS: rostral migratory stream
Sox2: sex determining region Y (SRY)‐box 2
SVZ: subventricular zone

## 1 Introduction

Since the beginning of the 20th century, it has been a long‐standing dogma that the adult nervous system does not produce new nerve cells after birth. The first groundbreaking discoveries to overturn this dogma were made by scientist Joseph Altman, who used radioactively labeled ^3^H‐thymidine to show potential new neurons in the nervous system of rodents and cats (Altman 1962; Altman and Chorover, 1963). Altman (1969) further investigated the characteristics and fate of cells in the subependymal layer of the anterior lateral ventricle of aging rats and its rostral extension to the olfactory bulb. He described this extension to the olfactory bulb as the so‐called “rostral migratory stream” (RMS). It was assumed that migration into the olfactory bulb results in renewal of its cells, while only a small proportion migrates into the anterior neocortex and the basal ganglia (Altman 1963; 1969). These previous studies have shown that there are specific migration pathways from neurogenic stem cell niches in the adult mammalian brain and that neurons can be replaced throughout life.

A few years later, newborn neurons were demonstrated in the dentate gyrus and olfactory bulb of adult rats (Kaplan and Bell 1983). Since then, the application of immunohistochemical methods and the use of 5‐bromo‐2′‐deoxyuridine (BrdU) as a thymidine analogue that is incorporated into actively dividing cells, has opened a growing field for the study of adult neurogenesis in different species, finally demonstrating its existence in the adult human hippocampus, while it was also showing that cells containing BrdU in the subventricular zone (SVZ) of the human caudate nucleus are still undifferentiated (Eriksson et al. 1998). Subsequently, based on its structural importance for learning and memory processes a large number of studies have focused on hippocampal adult neurogenesis (see Kempermann et al. 2004; Ming and Song 2011; Toda and Gage 2018; Denoth‐Lippuner and Jessberger 2021; Kempermann 2022 for review).

Only few however, studied adult neurogenesis in the mammalian cortex or other regions like the amygdala, the hypothalamus, the striatum and the substantia nigra (see Jurkowski et al. 2020 for review). In 2014, a study was published that again showed adult striatal neurogenesis in humans by using histological methods in combination with the carbon‐14 dating method, which in turn widened the field (Ernst et al. 2014; Kempermann 2014). Still, the phenomenon is understudied. Throughout time, there have been and still are controversies about adult neurogenesis, its meaning and importance that are justified either by the methods or materials used that show inconsistencies in quantity, but also because of the complexity of the subject, which makes it difficult to isolate certain related functions in the mammalian brain (Sorrells et al. 2018; Lee and Thuret 2018; Gage 2019; Boldrini et al. 2019; Leal‐Galicia et al. 2021; Franjic et al. 2022; Tosoni et al. 2023).

In parallel to the studies in mammals, Goldman and Nottebohm investigated adult neurogenesis in song birds and found a direct link between song development in canaries and newborn neurons close to and in a brain‐region called higher vocal center (HVC; Goldman and Nottebohm 1983). Although the study was of great interest, birds would not be considered as a general model organism to study the basic principles of adult neurogenesis. At the time limited knowledge of the avian brain and its functions existed and many researchers still thought that most parts of the bird's forebrain were striatal in nature. Decades later, this has proved to be a false assumption. Indeed, the largest parts of the avian forebrain are pallial in nature and showing that the avian brain is more comparable to mammalian brains than it was assumed (Reiner et al. 2004a; Jarvis et al. 2005). An increased number of studies in the avian species expanded the knowledge about adult neurogenesis in pallial structures comparable to the neocortex or the hippocampus, and few included subpallial structures like the basal ganglia and diencephalic structures like the hypothalamus, and the potential role of adding newborn neurons to existing networks for learning, memorizing, song production, food‐hoarding and migration/navigation (Alvarez‐Buylla and Nottebohm 1988; Barnea and Nottebohm 1994; Patel et al. 1997; Scharff et al. 2000; Doetsch and Scharff 2001; Gahr et al. 2002; Hoshooley and Sherry 2007; Thompson et al. 2007; Pravosudov and Smulders 2010). As a result, Barnea and Pravosudov first mentioned that birds are a highly suitable model to study adult neurogenesis in general (Barnea and Pravosudov 2011). Over the last decade, knowledge of the avian brain has increased enormously, showing not only that birds have similar cognitive functions comparable to mammals, but also that the network architecture underlying these functions is similar (Emery and Clayton 2005; Herold et al., 2011; Clayton and Emery 2015; Olkowicz et al. 2016; Stacho et al. 2020; Nieder et al. 2020; Güntürkün et al. 2024, 2021). In addition, more detailed studies of avian adult neurogenesis emerged, indicating that the long‐time generation of new neurons all over the forebrain is absolutely essential for birds and that it follows specific patterns depending on environments, stress, sex, regulation of song behavior, sociality, seasonality, and modulating bird song (Melleu et al. 2013; Brenowitz and Larson 2015; Balthazart and Ball 2016; Mazengenya et al. 2018, 2017; Robertson et al. 2017; Herold et al. 2019; Mehlhorn et al. 2022; Brenowitz et al. 2024). Yet, none of the studies focused on the quantification of striatal adult neurogenesis in birds nor compared it directly with one of the most studied mammalian model organism, the mouse.

**FIGURE 1:**
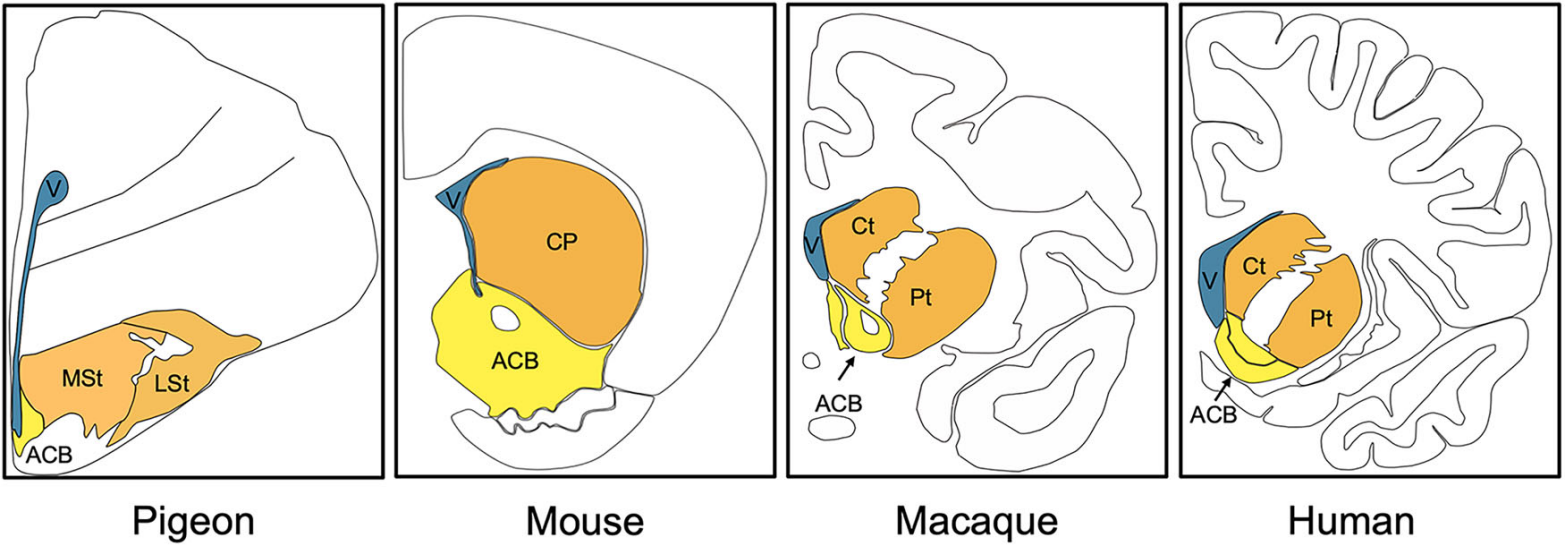
Schematic overview of striatal regions of pigeon, mouse, macaque and human. The dorsal striatum is shown in orange, the ventral striatum in yellow, and the ventricles (V) in blue. In the pigeon brain, the medial striatum (MSt), lateral striatum (LSt), and nucleus accumbens (ACB) are indicated. In the mouse brain, the caudoputamen (CP) and nucleus accumbens (ACB) are labeled. In the macaque and human brains, the caudate nucleus (Ct), putamen (Pt), and nucleus accumbens (ACB) are shown. In all species the subpallial striatum develops from the same origin, the basal telencephalic anlage that includes three major radially oriented histogenetic zones, and then undergoes species‐specific structural adaptations. The coronal slides are not scaled.

Birds and mammals show remarkable similarities in the development of the subpallium (Puelles et al. 2000; Stühmer et al. 2002; Cobos et al. 2005; Long et al. 2009; Abellán and Medina 2009; Kuenzel et al. 2011; Medina et al. 2014; Rueda‐Alana et al. 2025; Hecker et al. 2025). In general, the striatum of birds consists of the medial striatum (MSt), the lateral striatum (LSt), the intermediate striatum (ISt, formerly known as intrapeduncular nucleus, INP) and the nucleus accumbens (ACB; Figure 1), while some birds show species‐specific functional and structural adaptations in parts of this regions (Jarvis et al. 2013; Bruce et al. 2016). Thereby, MSt and LSt share traits with the dorsal striatum and thus the caudoputamen (CP) of rodents, including a neuropil rich in acetylcholinesterase (AChE), dopamine, substance P, and enkephalin and high densities of dopamine and muscarinic receptors (Kuenzel et al. 2011; Herold et al. 2018). Further, both, MSt and LSt show similar connectivity patterns to CP, and based on the afferent and efferent connections can be further subdivided, making them functionally comparable to the limbic striatum, associative CP, or somato–motor striatum (Veenman et al. 1995; Reiner 2002; Reiner et al. 2004b; Güntürkün 2005; Kuenzel et al. 2011; Jarvis et al. 2013; Letzner et al. 2016; Bruce et al. 2016; Steinemer et al. 2024). The caudal‐ and lateralmost part of the LSt shows a distinct and different neuropil than the mammalian striatum and is likely part of the central extended amygdala, containing cells projecting to the hypothalamus (Jarvis et al. 2013; Vicario et al. 2014; Bruce et al. 2016).

The formerly named INP occupies a special position in the bird's striatum. Originally classified as a pallidal region, it was increasingly recognized that it has predominantly dorsal striatal characteristics with a small number of cells that may have migrated from pallidal regions during its development and thus should be referred to as ISt (Reiner et al. 2004b; Jarvis et al. 2013; Bruce et al. 2016).

The ACB is a part of the ventral striatum in birds and mammals (Reiner et al. 2004a; Bálint and Csillag 2007; Balint et al. 2011; Bruce et al. 2016). In mammals, the ACB is divided into core and shell regions based on its neurochemical properties, with the shell region often being further divided into medial and lateral parts (Floresco 2015). Comparable segmentations were reported for birds (Bálint and Csillag 2007; Balint et al. 2011; Kuenzel et al. 2011; Bruce et al. 2016). Altogether, striatal connectivity of birds resembles the typical cortico–striatal–thalamic loops of mammals, which makes them an interesting model to study the fundamentals of striatal adult neurogenesis (Graybiel 2000; Doupe et al. 2005; Gale and Perkel 2010; Güntürkün et al. 2024).

Comparable to mammals, in birds, striatal subregions are in close proximity to the SVZ, which holds the potential stem cell niches to generate new neurons over lifespan. Thus, the SVZ appears to play an important role from which new neurons potentially emerge and migrate into the striatum. However, rodents and birds show a different architecture of the SVZ compared to each other, as well as to primates (Alvarez‐Buylla et al. 1998; Doetsch et al. 1999; Doetsch and Scharff 2001; Quinones‐Hinojosa et al. 2006; Gil‐Perotin et al. 2009; Sawamoto et al. 2011; Melleu et al. 2013; Inta et al. 2015; Mehlhorn et al. 2022). In birds, all along the ventricles SVZ stem cell niches and cell division can be detected with some “hot spots” along the anterior–posterior axis in the ependyma of the ventricles (Alvarez‐Buylla and Nottebohm 1988; Alvarez‐Buylla et al. 1998; Mezey et al. 2012; Melleu et al. 2013; Brenowitz and Larson 2015; Mehlhorn et al. 2022). In pigeons, two of these “hot spots” directly neighbor MSt/ACB (anterior) and LSt (posterior; Melleu et al. 2013; Mehlhorn et al. 2022). Nevertheless, along with adult neurogenesis, cells pass from the neurogenic niches through various stages up to maturation and migrate to their final destination. This process is comparable over the different species. The neurogenic niche(s) in the lateral wall of the lateral ventricle contain a subpopulation of cells known as B‐cells or SVZ astrocytes that are multipotent stem cells. These stem cells have the potential to develop into both, neurons and glial cells (Reynolds and Weiss 1992; Doetsch et al. 1999; Seri et al. 2001; Kriegstein and Götz 2003; Casper and McCarthy 2006). At the first stage, the B‐cells in the SVZ are activated and generate type C‐cells via asymmetric cell division. Type B‐cells express markers like GFAP, (sex determining region Y)‐box 2 (Sox2), epidermal growth factor receptor (EGFR), Mash1, and Nestin. As they are proliferatively active, endogenous proliferation markers such as Ki67 or proliferating cell nuclear antigen (PCNA) can be detected in B‐cells (Doetsch et al. 1997; Pastrana et al. 2009; Kim et al. 2011; Zhang and Jiao 2015; Niklison‐Chirou et al. 2020; Mehlhorn et al. 2022). The C‐cells comprise the second stage of development and are GFAP‐negative but Sox2‐positive and proliferate increasingly and highly express Ki67 or PCNA. They still express Nestin, EGFR, Mash1 and additionally, the transcription factor homeobox protein Dlx‐2 (Doetsch et al. 1997; Pastrana et al. 2009; Kim et al. 2011; Zhang and Jiao 2015; Niklison‐Chirou et al. 2020). The third stage comprises type A‐cells that develop from C‐cells. A‐cells are characterized by the expression of polysialylated form of the neural cell adhesion molecule (PSA‐NCAM), Dlx‐2, and DCX. These cells can be distinguished from C‐cells, as they are DCX‐positive and Nestin‐negative that indicates an increasing neuronal differentiation (Bonfanti and Theodosis 1994; Brown et al. 2003; Jones and Connor 2012; Zhang and Jiao 2015; Niklison‐Chirou et al. 2020; Mehlhorn et al. 2022). To reach the fourth stage, the cells increasingly differentiate into mature neurons. At this stage the transition from early neuronal maturation to fully differentiated neurons can be identified by marker combinations with DCX, Neuronal nuclei marker (NeuN) and calretinin that are sometimes coexpressed (Ernst et al. 2014; Zhang and Jiao 2015; Gusel'nikova and Korzhevskiy 2015; Petryszyn et al. 2018; Mehlhorn et al. 2022). Finally, in the fifth stage neurons are matured and express NeuN and other typical neuronal markers, such as calbindin, calretinin, and choline acetyltransferase that can indicate the integration into the striatal neuronal network. This marks the completion of neurogenesis (Baimbridge et al. 1992; Zhang and Jiao 2015; Gusel'nikova and Korzhevskiy 2015; Petryszyn et al. 2018). Thus to gain more fundamental knowledge of the potential of striatal adult neurogenesis over different species, including primates, the composition of expression markers in the SVZ and the architecture of the SVZ should be of interest.

Here, we systematically investigated striatal adult neurogenesis in pigeons (*Columba livia* f.d.), mice (*Mus musculus*), macaques (*Macaca fascicularis*), and in an exemplary, individual human (*Homo sapiens*) brain. To achieve this, we first provide a quantitative analysis of neuronal precursor cells and immature neurons with the neurogenic marker DCX, adult‐born matured neurons with the combination of BrdU and NeuN, and adult‐born glia with the combination of BrdU and GFAP in the main striatal subregions of pigeons and mice. Second, the proliferative potential of cells in the SVZ proximal to the caudate nucleus of macaques and human were analyzed in terms of the cellular architecture and the presence of the neuronal/glial precursor and proliferative markers Sox2, GFAP and Ki‐67, DCX, Dlx‐2, NeuN, and Calretinin in the SVZ and close neighborhood. The overall goal for each analysis was to gain a deeper understanding of differences and similarities between species to encourage the use of different species to study adult striatal neurogenesis in the future.

## 2 Material and Methods

### 2.1 Subjects

#### 2.1.1 Pigeons and Mice

Nine adult Homing pigeons (*Columba livia* f.d, four females, five male) and seven adult mice (*Mus musculus*, C57BL/6, all male) were used for this study. The pigeons were kept in an open‐air laboratory of the Heinrich Heine University and had the opportunity to leave the loft and fly freely. They had ad libitum access to food and water as well as grit and minerals. The mice were kept in the animal housing facilities of the University Hospital Düsseldorf (*ZETT*). They were housed conventionally under SPF conditions in two groups of five mice in Makrolon type III cages (37 × 22 × 15 cm). Mice were kept in an enriched environment (EE) with toys, nest‐building material and a running wheel. The EE changed weekly. The light–dark rhythm was 12 h. Food and water were available ad libitum in this housing system.

At the start of the experiment, adult pigeons were 12‐month‐old and adult mice 9 weeks. Both species received 50 mg/kg BrdU (Sigma‐Aldrich, Germany) injections on three consecutive days (mice i.p., pigeons i.m.) and were further kept as described above. Pigeons were sacrificed 3 months later and mice 2 months later with an overdose of Pentobarbital (70 mg/mL). Both species were transcardially perfused with 0.9% saline and 4% paraformaldehyde (PFA) for optimal immunohistochemical conditions. Brains were removed, stored overnight in postfixative (4% PFA, 15% sucrose) at 4°C, placed in a 30% sucrose phosphate buffered saline solution the following day for cryoprotection and then stored at −80°C until processing.

All applicable international, national, and/or institutional guidelines for the care and use of animals were followed. The study was approved by the Ethics Committee of Animal Welfare of the state of North Rhine‐Westphalia (*LANUV*), Germany (Ref. 84‐02.04.2014.A345 (pigeons); Ref. 87‐51.04.2010.A250 (mice)).

#### 2.1.2 Macaques

Five adult (6 years old) Macaque (*Macaca fascicularis*, four females, one male) brains were provided from Covance Laboratories (Münster, Germany) in the time between 2000 and 2002. The fresh brains were cut into 1 cm thick slices, immersion‐fixed in Zamboni fixative solution at pH7.4 (Bidmon et al. 2006) for 3 days at 4°C with constant agitation. After that the slices were cut into blocks and all tissues were transferred into 25% sucrose phosphate buffered saline for cryoprotection. After sinking, they were frozen and stored at −80°C for later use. Here, only the blocks containing striatal regions and the SVZ of both hemispheres were studied. All procedures followed in accordance with the guidelines of the European Communities Council Directive for the care and use of animals for scientific purposes within the Directive of 2002.

#### 2.1.3 Human

One human brain was obtained from the body donor program at the medical faculty of the Heinrich Heine University (ethics approval no. 2023‐2632). It was a female donor who died of heart failure at the age of 83. The post mortem interval was 20 h and the total brain weight was 1155 g. The fresh brain tissue was cut into 13 coronal slices, each 1 cm thick, in each hemisphere and then immersion‐fixed in formalin pH 7.4 at 4°C with constant agitation for several weeks. Subsequently, each slice was divided into smaller blocks, placed in a 30% sucrose solution for cryoprotection and then stored at −80°C until further processing. Again, only the blocks containing striatal regions and the SVZ of both hemispheres were used.

### 2.2 Immunohistochemistry

Either the whole brain (pigeons and mice) or the striatal blocks (macaque and human) were cut into series of ten using a cryostat microtome (Leica SM200R/Reichert‐Jung, Germany). Coronal Sections of 40 µm (pigeon, macaque, human) and 32 µm (mouse) along the anterior‐posterior axis were prepared, and as such each series contained a number of slices at a distance of 400 or 320 µm, respectively. The slices were kept free‐floating at 4°C in a solution of 0.12 M PBS and 0.3% sodium azide until immunohistochemical processing.

For the analysis of adult striatal neurogenesis, the following primary antibodies were used (see Table 1).

**TABLE 1:** Overview of primary antibodies used to analyze adult striatal neurogenesis in different species (p, pigeon; ma, macaque, mo, mouse, h, human).

Antibody	RRID	Company	Dilution	Species
Anti‐BrdU, Rat, monoclonal OBT 0030	AB_609568	AbD Serotec, United Kingdom	1:200	p, mo
Anti‐DCX, Rabbit, polyclonal, ab18723	AB_732011	Abcam, United Kingdom	1:500	h, ma, mo, p
Anti‐NeuN, Mouse, monoclonal MAB377	AB_2298772	Merck Millipore, United States	1:1000	h, ma, mo, p
Anti‐GFAP, Rabbit, polyclonal, Sigma G9269	AB_477035	Sigma‐Aldrich, United States	1:500	p, mo
Anti‐Calretinin, Guinea‐pig, polyclonal, ABIN 1742427	—	Antibodies‐Online, Germany	1:1000	h, ma
Anti‐GFAP, Chicken, polyclonal, AB5541	AB_177521	Merck Millipore, United States	1:500	h, ma
Anti‐Sox2, Mouse, monoclonal, sc365823	AB_10842165	Santa Cruz, Europe	1:200	h, ma
Anti‐Ki67, Rabbit, polyclonal, ab15580	AB_443209	Abcam, United Kingdom	1:200	h, ma
Anti‐Dlx‐2, Rabbit, polyclonal, PA5‐40505	AB_2608066	Invitrogen, United States	1:200	h

The detailed processing of free‐floating sections has already been described (Herold et al. 2019; Mehlhorn et al. 2022). An overview of the individual washing/rinsing steps is provided (Supporting Information Table 1). Separate brain section series from a total of nine pigeons (*N* = 9) were treated with BrdU/NeuN/GFAP triple labeling (*N* = 9 pigeons) or BrdU/DCX double labeling (*N* = 9 pigeons). Separate brain section series from a total of seven mice (*N* = 7) were treated with BrdU/NeuN/GFAP/Hoechst fourfold labeling (*N* = 5 mice) or BrdU/DCX/Hoechst triple labeling (*N* = 5 mice). Separate macaque (*N* = 5) and human (*N* = 1) striatal brain block section series were treated with GFAP/Sox2/Ki‐67/DAPI fourfold staining or DCX/NeuN/Calret/DAPI fourfold staining. In addition, double labeling for DCX/DAPI and Dlx‐2/DAPI was used to clearly identify further cell types and stages of adult neurogenesis in the human caudate nucleus. The used secondary antibodies in combination with different fluorescent dyes and specific dilutions are provided in Table 2. In general, DAPI and Hoechst served as nuclear staining dyes.

**TABLE 2:** List of secondary antibodies used in different combinations for specific binding of primary antibodies and fluorescent microscopy (p, pigeon; ma, macaque, mo, mouse, h, human).

Antibodies	RRID	Company	Dilution	Species
Donkey anti‐Rabbit‐ Alexa Fluor 488, 711‐545‐152	AB_2313584	Jackson ImmunoResearch, United Kingdom	1:200	h, ma
Donkey anti‐Mouse‐ Alexa Fluor 647, 715‐607‐003	AB_2340867	Jackson ImmunoResearch, United States, Dianova, Germany	1:200	h, ma, mo, p
Donkey anti‐Guinea‐Pig Cy3, 706‐165‐148	AB_2340460	Jackson ImmunoResearch, Europe	1:200	h, ma,
Donkey anti‐Rabbit‐Alexa Fluor 647, 715‐605‐152	AB_2492288	Jackson ImmunoResearch, United States	1:200	h
Goat anti‐Chicken‐ Alexa Fluor 488, 103‐545‐155	AB_2337390	Jackson ImmunoResearch, United States	1:200	h, ma
Goat anti‐Mouse‐ Alexa Fluor 647 115‐605‐003	AB_2338902	Jackson ImmunoResearch, United States	1:200	h, ma
Goat anti‐Rabbit‐ Cy3, 111‐165‐003	AB_2338000	Jackson ImmunoResearch, United States, United Kingdom	1:200	h, ma, mo, p
Goat anti‐Rabbit‐ FITC, 10006588	AB_10097845	Cayman Chemical, United States	1:200	p, mo
Goat anti‐Rat‐ CY3, AP183C	AB_92596	Merck Millipore, United States	1:200	p, mo
Goat anti‐Rat‐ Alexa Fluor 488, 112‐545‐003	AB_2338351	Jackson Immuno‐Research, United Kingdom	1:200	p, mo

All antibodies were validated by control rounds without the primary or the secondary antibody, western blotting procedures or confirmed specific binding in previous studies (Herold et al. 2019; Mehlhorn et al. 2022).

### 2.3 Data Analysis

Stained sections from all species were digitized at 20 × magnification in a fluorescence microscope (AxioScan.Z1, Zeiss, Germany or Olympus VS200, Olympus, Germany).

For the quantitative analysis in pigeons and mice, the relevant areas of the striatum along the anterior–posterior axis were segmented in all sections in the left hemisphere and their area (in mm^2^) was estimated using the ZEN software (Zeiss, Germany). For each section the corresponding atlas‐level was determined. Thereby, the brain regions and atlas planes were identified using “The stereotaxic atlas of the brain of the pigeon” (Karten and Hodos 1967) and the nomenclature recommended by the “Avian Brain Nomenclature Forum” (Reiner et al. 2004a) as well as the findings from the study by Bruce et al. (2016) and Jarvis et al. (2013). For the mouse brains, the “Allen Mouse Brain Reference Atlas” (Lein et al. 2007; http://mouse.brain‐map.org) was used as a reference for the identification of the brain regions and atlas levels. In pigeons, the ACB, MSt, LSt, ISt and globus pallidus (GP), and in mice CP, fundus striatum (FS), ACB and GP were analyzed. The signals of the immunoreactive cells were counted manually in all delineated striatal areas in each slice along the anterior–posterior axis with the following markers and marker combinations: BrdU+, DCX+, BrdU+/NeuN+, BrdU+/GFAP+, and BrdU+/DCX+ by using ZEN. Thus, a mean value for each region based on a number of coronal slices from all positions of striatal regions along the anterior–posterior axis (number depends on the size of the striatal structure) of each animal. As such the number of immunoreactive cells per mm^2^ was determined for each animal per region. Because GFAP also marks stem cells, we cannot completely exclude that few of the BrdU/GFAP cells in the parenchyma counted are stem cells, although the shape of potential progenitors that express GFAP and might still develop into neurons usually differs compared to matured glial cells and are often organized in triades or detectable within the proliferative zones of the VZ (the zone was not included into our counting, Mehlhorn et al. 2022).

The macaque and human slides were qualitatively inspected and analyzed for signs of adult striatal neurogenesis. For the human brain, sections and structures were identified with data from the Julich‐Brain atlas (https://atlases.ebrains.eu/viewer/go/julichbrain), while for the macaque brains the multilevel‐macaque brain atlas (Balan et al. 2024) and Paxinos et al. (2000) were utilized. The focus here was a comprehensive analysis of the SVZ close to the caudate nucleus of both species to explore the layered cytoarchitecture and identify stem and progenitor cells in this specific region to gain more information about the potential for adult striatal neurogenesis. For this purpose, the biomarkers Sox2, GFAP, and Ki67 were used to label specific cell types within a stem cell niche (Doetsch et al. 1997; Gil‐Perotin et al. 2009; Quinones‐Hinojosa et al. 2006). To further identify different cellular localization of the respective labels, and to exclude false signals, DAPI was used to mark the cell nuclei. Within our cytoarchitectonical analysis we followed the classification of the human and macaque SVZ by Quinones‐Hinojosa et al. (2006) and Gil‐Perotin et al. (2009). In addition, individual slices from different series were stained with DCX/NeuN/Calret/DAPI and investigated to show the possible occurrence of proliferative cells in comparison to matured neurons. Further, double labeling for DCX/DAPI and Dlx‐2/DAPI was used to identify stages of adult neurogenesis in the caudate nucleus as a candidate for either migrating or adding newborn neurons over lifetime (Ernst et al. 2014).

### 2.4 Statistical Analysis

The mean values of the individually measured signals/mm^2^ per subregion along several sections of the anterior–posterior axis of the pigeon and mouse striatum were calculated for each animal individually. Then mean values and standard error means per subregion were calculated (pigeon *N* = 9 (all staining procedures); mouse *N* = 5 (different groups of animals for specific staining procedures)). This enabled a comparison of the individual markers in the respective subregions of mice and pigeons.

A nonparametric analysis of variance, the Friedman test, was initially used for the statistical tests between the different subregions and markers for each species separately. If the Friedman‐ANOVA (FRM ANOVA) showed significant differences, the Wilcoxon signed‐rank test (*W*‐test) was used for pairwise comparison of the subregions. For the analysis between the two species (pigeon and mouse), a Mann–Whitney *U* (MWU) test was carried out for pairwise comparison of regions between groups. Initially, the level of significance was set at *p* < 0.05. Then, for all post hoc tests the Benjamini–Hochberg correction was used to avoid alpha error accumulation in multiple tests. The same method was applied for the Mann–Whitney *U* approach between species. Only those results are reported that still proofed significance after *p*‐level correction. The statistical tests were carried out using the SPSS program from IBM (version 29).

## 3 Results

### 3.1 Overview of Different Stages of Adult Neurogenesis in the Striatum of Pigeons and Mice

**FIGURE 2:**
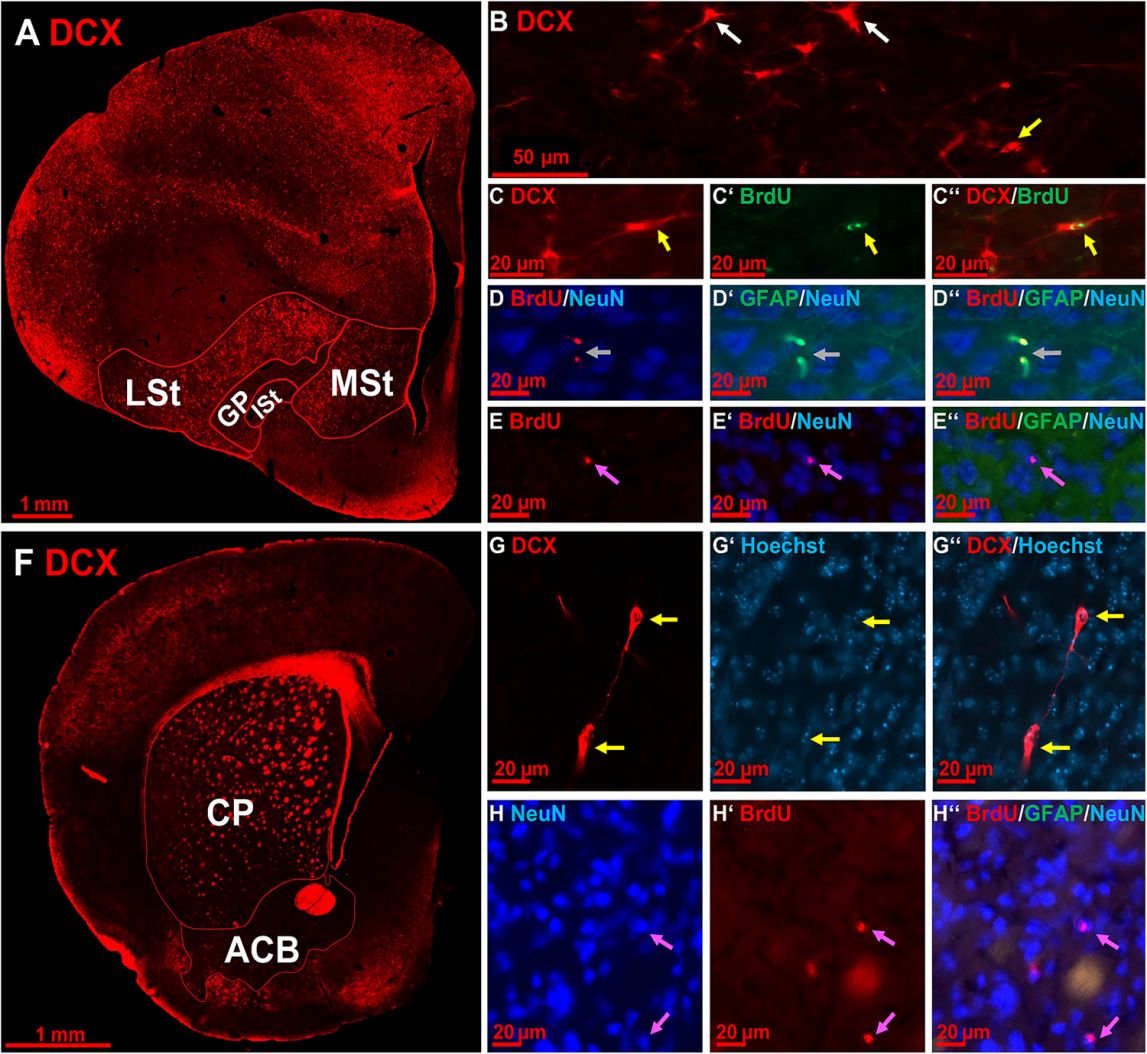
Striatal adult neurogenesis in pigeons and mice. In pigeons, DCX+ cells and fibers in striatal areas (A) magnified in (B) show different triangular (white arrows) and ovoidal (yellow arrow) shapes. Three months after injection of BrdU, BrdU+/DCX+ cells were still detectable underlying that adult neurogenesis is not fully completed in all neurons holding a reserve (C). BrdU+/GFAP+ cells demonstrate gliogenesis, while adult born, matured neurons were tracked with BrdU+/NeuN+ signals (D, E). In mice, Doublecortin (DCX)+ cells the striatal areas (F) emerged as ovoidal (G). Two months after injection of BrdU, adult born, and matured neurons were identified with BrdU+/NeuN+ signals (H). The globus pallidus (GP) was additionally investigated as part of the basal ganglia. ACB, nucleus accumbens; CP, caudoputamen; GP, globus pallidus; ISt, intermediate striatum; LSt, lateral striatum; MSt, medial striatum.

**FIGURE 3:**
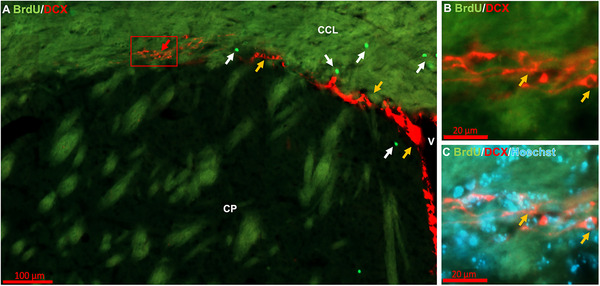
Detail of the rostral migratory stream (RMS) adjacent to the mouse striatum. BrdU/DCX/Hoechst labeled brain section of the caudoputamen (CP), adjacent to the ventricle (V), and corpus callosum (CCL) showing labeling in the RMS, with white arrows marking BrdU+ cells and orange arrows marking DCX+ cells. In B and C enlargement of the box in A shows the ovoidal shape of DCX+ cells in the RMS.

Different stages of adult neurogenesis and newborn neurons were identified in the striatum of pigeons and mice (Figure 2). DCX+ cells were subdivided into DCXov+ (migrating neurons, ovoidal soma with two stronger processes), DCXtr+ (neurons in the stadium of differentiation/integration) and DCX+/BrdU+ (newborn, not fully matured neurons) cells (Figure 2A–C). In addition, NeuN+/BrdU+ (matured/integrated adult newborn neurons) and GFAP/BrdU+ (adult newborn glia) cells were identified in the pigeon proving active adult neuro‐ and gliogenesis in the striatum of pigeons (Figure 2D,E). In mice, the few detected DCX+cells predominantly had an ovoidal structure, often with a pronounced protrusion, while neither DCXtr+ nor DCX+/BrdU+ cells were observed (Figure 2F,G). Additionally, few NeuN+/BrdU+ (matured/integrated adult newborn neurons) cells were detected, but no GFAP/BrdU+ cells although GFAP+ cells (glia) were present (Figure 2H). More generally, in mice, DCX+ and BrdU+ cells were primarily localized in the periphery of the CP along the corpus callosum (CC) and the RMS (Figure 3A–C).

### 3.2 Quantitative Analysis of Adult Neurogenesis in the Striatum of Pigeons and Mice

#### 3.2.1 Pigeon

**FIGURE 4:**
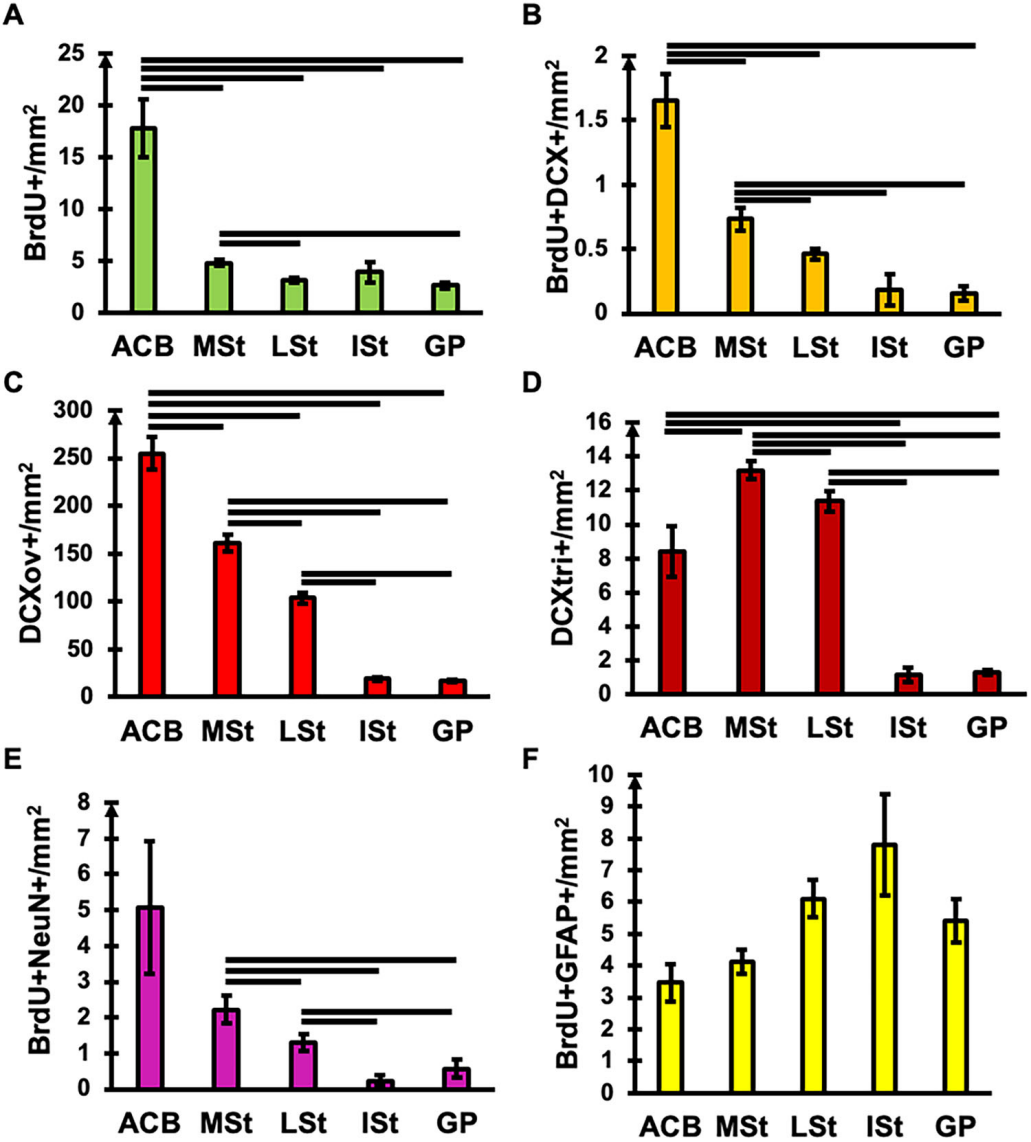
Quantitative analysis of neurogenic cells in striatal regions of the pigeon. The distribution of BrdU+ (A), BrdU+/DCX+ (B), DCXov+ (C), DCXtri+ (D), BrdU+/NeuN+ (E), and BrdU+/GFAP+ cells/mm^2^ is presented for the nucleus accumbens (ACB), medial striatum (MSt), lateral striatum (LSt), intermediate striatum (ISt), and globus pallidus (GP). The globus pallidus (GP) was additionally investigated as part of the basal ganglia. The columns show mean values ± standard error. The horizontal lines show significant differences between the areas (Wilcoxon signed‐rank test, Benjamini–Hochberg correction, all *p* < 0.05).

The quantitative analysis of BrdU/DCX and BrdU/GFAP/NeuN staining of the pigeon showed a differential expression pattern in the ACB, MSt, LSt, ISt, and GP (Figure 4).

Significant regional differences were found for BrdU/DCX signals [FRM ANOVA, BrdU+: χ^2^(*n* = 9, df = 4) = 26.40, *p* < 0.001; DCXov+: χ^2^(*n* = 9, df = 4) = 33.51, *p* < 0.001; DCXtri+: χ^2^(*n* = 9, df = 4) = 31.56, *p* < 0.001; BrdU+/DCX+: χ^2^(*n* = 9, df = 4) = 25.74, *p* < 0.001; Supporting Information Table 2].

The highest numbers of BrdU+ cells were detected in the ACB (Wilcoxon signed‐rank test Benjamini–Hochberg correction; all *p* < 0.05; Figure 4A). In addition, the number of BrdU+/DCX+ immature neurons in the ACB were higher compared to the MSt, LSt and GP (Wilcoxon signed‐rank test Benjamini–Hochberg correction; *p* < 0.05; Figure 4B). The MSt showed the second highest counts for BrdU+/DCX+ signals and differed from the LSt and GP as well as from the ACB (Wilcoxon signed‐rank test Benjamini–Hochberg correction; all *p* < 0.05; Figure 4B and Supporting Information Table 2).

The ACB showed again differences in the number of DCXov+ signals if compared to all other subregions (Wilcoxon signed‐rank test Benjamini–Hochberg correction; *p* < 0.05; Figure 4C) and expressed the highest counts for ovoidal DCX+ cells (Supporting Information Table 2).

The MSt showed the second highest number of DCXov+ signals compared to the other areas (Wilcoxon signed‐rank test; all *p* < 0.05), followed by the LSt (Wilcoxon signed‐rank test Benjamini–Hochberg correction; all *p* < 0.05; 4C, Supporting Information Table 2). Analysis of the DCXtri+ cells also revealed significant differences between the MSt and the ACB and between the MSt and the LSt and all other areas (Wilcoxon signed‐rank test Benjamini–Hochberg correction; *p* < 0.05; Figure 4D), with the MSt holding the highest number of DCXtri+ signals, followed by the LSt (Figure 4D and Supporting Information Table 2).

General regional differences were determined for the BrdU/GFAP/NeuN analysis [FRM ANOVA, BrdU+: χ^2^(*n* = 9, df = 4) = 16.98, *p* < 0.05; BrdU+/GFAP+: χ^2^(*n* = 9, df = 4) = 11.73, *p* < 0.05; BrdU+/NeuN+: χ^2^(*n* = 9, df = 4) = 17.30 *p* < 0.05; Figure 4E,F and Supporting Information Table 3]. Again, the ACB showed a higher number of BrdU+ signals compared to the other areas (Wilcoxon signed‐rank test Benjamini–Hochberg correction; all *p* < 0.05; Supporting Information Table 3).

In addition, the MSt and LSt showed a significantly higher number of BrdU+ cells compared to GP (Wilcoxon signed‐rank test Benjamini–Hochberg correction; both, *p* < 0.05). In terms of the number of BrdU+/NeuN+ signals, the MSt and LSt showed differences compared to the ISt and GP, with the MSt showing more BrdU+/NeuN+ neurons than the LSt, ISt and GP, followed by the LSt (Wilcoxon signed‐rank test Benjamini–Hochberg correction; all *p* < 0.05, Figure 4E and Supporting Information Table 3). No differences between regions after statistical correcting procedures were detectable for the number of BrdU+/GFAP+ signals (Figure 4F). Further, the analysis of striatal regions for specific positions along the anterior–posterior axis did not result in any consistent effects (Supporting Information Figures 1 and 2).

#### 3.2.2 Mouse

**FIGURE 5:**
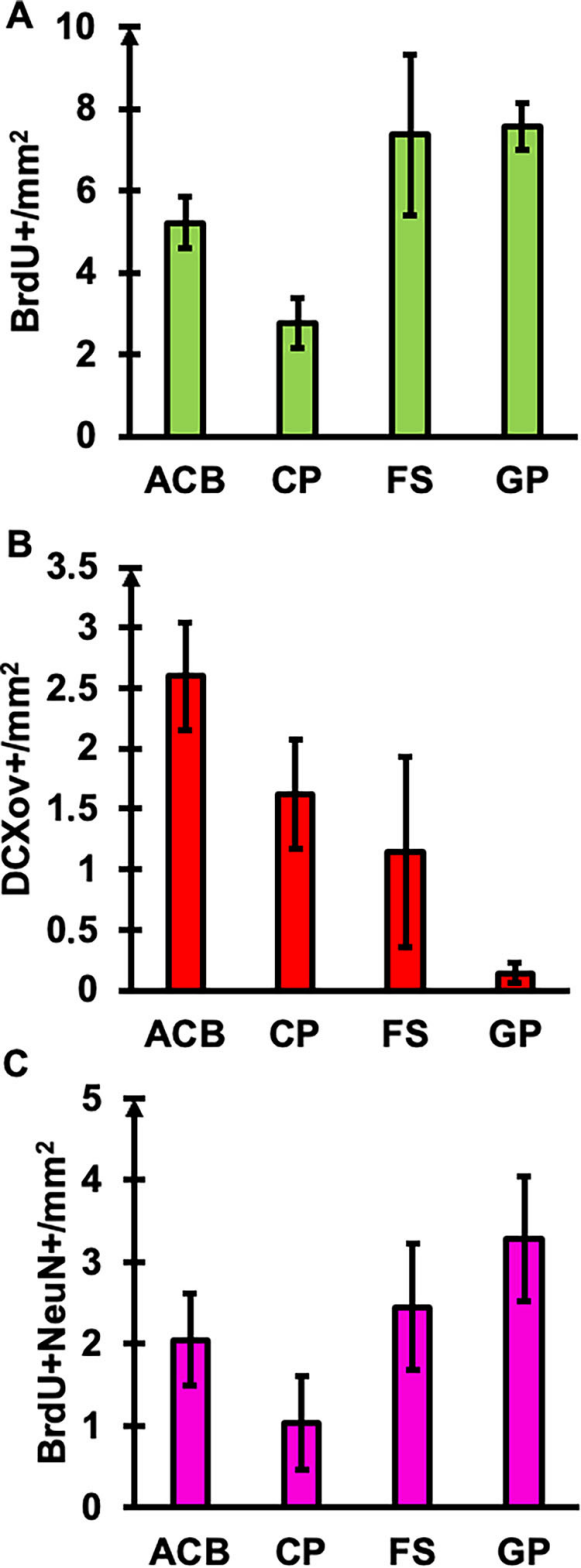
Quantitative analysis of neurogenic cells in striatal regions of the mouse. The distribution of BrdU+ (A), DCXov+ (B), and BrdU+/NeuN+ (C) cells/mm^2^ is presented for the nucleus accumbens (ACB), caudoputamen (CP), fundus striatum (FS) and globus pallidus (GP). The globus pallidus (GP) was additionally investigated as part of the basal ganglia. The columns show mean values ± standard error. No significant differences were detected between the studied areas.

Overall, the quantitative analysis of mouse striatal regions revealed only low levels of adult neurogenesis, while gliogenesis as measured by BrdU+/GFAP+ signals was completely absent. First, only low levels of BrdU+ and DCXov+ were detected in the CP, ACB, FS and GP (Figure 5A,B and Supporting Information Table 4) and no significant differences between subregions were observed. Second, DCXtri+ or BrdU+/DCX+ cells were not observed in any analyzed subregion of the mouse striatum.

The results for the BrdU/GFAP/NeuN analysis were similar. Only a low number of BrdU+ and BrdU+/NeuN+ cells was measured in subregions of the mouse striatum that showed no significant differences (Figure 5C and Supporting Information Table 5). No BrdU+/GFAP+ signals were observed in any of the analyzed striatal subregions.

#### 3.2.3 Pigeons Compared to Mice

**FIGURE 6:**
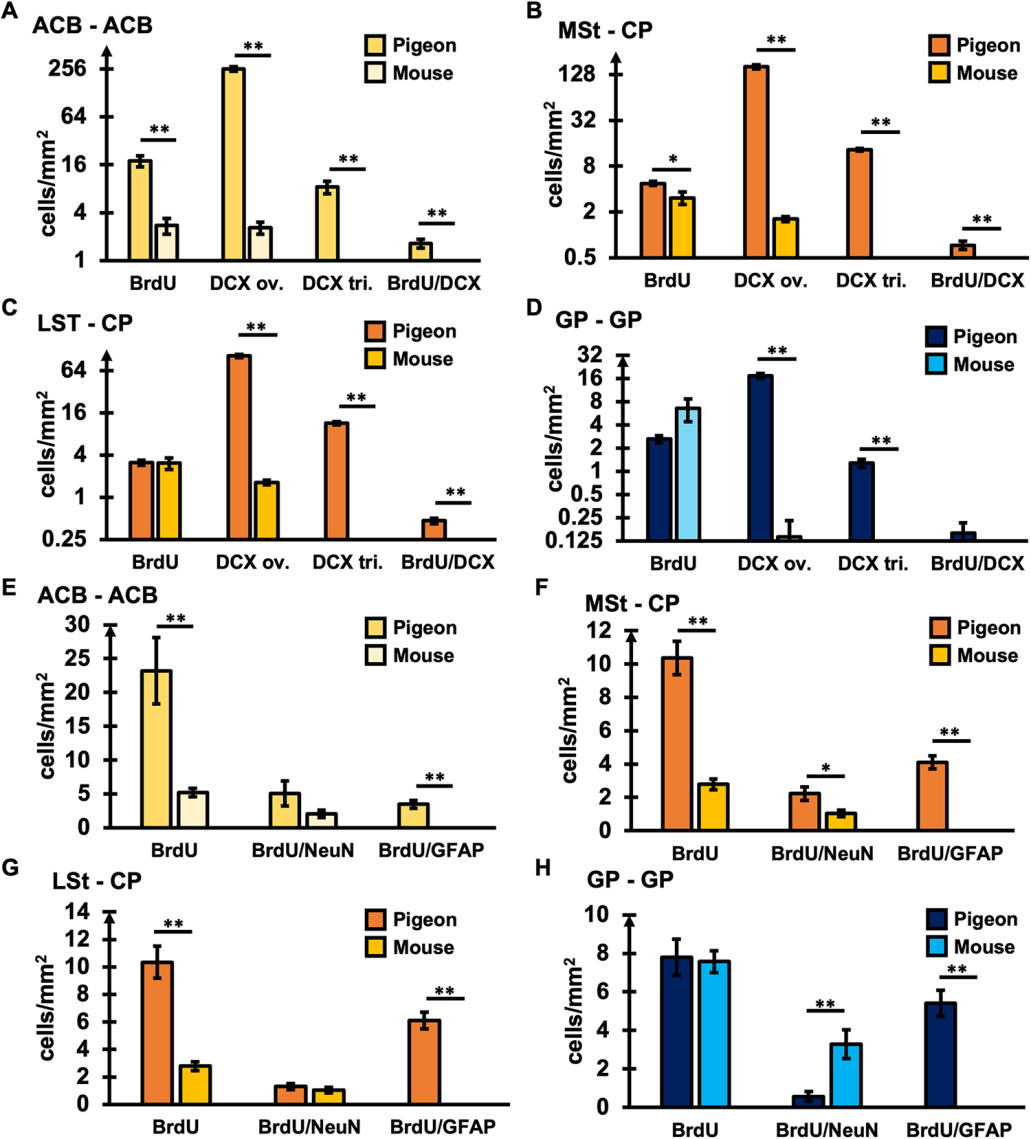
Comparison of striatal adult neurogenesis between pigeons and mice. In A‐D the distribution of BrdU+, DCXov+, DCXtri+ and BrdU+/DCX+ cells in pigeons and mice in (A) the nucleus accumbens (ACB), (B) the medial striatum (MSt) and caudoputamen (CP), (C) the lateral striatum (LSt) and CP and (D) the globus pallidus (GP) is shown. In E–H the distribution of BrdU+, BrdU+/NeuN+, and BrdU+/GFAP+ cells in pigeons and mice in (E) the ACB, (F) the MSt and CP, (G) the LSt and CP, and (D) the GP is presented. The globus pallidus (GP) was additionally investigated as part of the basal ganglia. All columns show mean values ± standard error. The horizontal lines show significant differences between the areas (Mann–Whitney *U*‐test, Benjamini–Hochberg correction *p < 0.05, **p < 0.01). (A–D) Note the logarithmic scaling of the *y*‐axis: log_2_ (*x*).

To highlight the differences in striatal neurogenesis between pigeons and mice, functionally equivalent subregions of the two species were compared for quantitative analysis. Thereby the medial (MSt) and lateral (LSt) striatum of the pigeon, were individually compared to CP of the mouse. In addition, the ACB and GP were analyzed (Figure 6). All measurements were included, even those that showed no signals in mice and as such, no signs of immature, nondifferentiated/integrated neurons (DCXtr+) or adult‐born glial cells (BrdU+/GFAP+).

Considerable differences in the amounts of migrating or immature neurons in striatal regions of the two species were detected (Figure 6A–D). In particular, significant differences were found in the ACB for all markers [all MWU; BrdU+ or DCXov+: both, *Z* = −3, *p* = 0.003; DCXtr+: *Z* = −3.068, *p* = 0.003; BrdU+/DCX+: *Z* = −3.072, *p* = 0.003, Benjamini–Hochberg correction; Figure 6A]. Independent of the stage, all levels of neurogenesis were higher in pigeons compared to mice. Similar results were obtained for the comparison between MSt and CP [all MWU; BrdU+: *Z* = −2.067, *p* = 0.039; DCXov+: *Z* = −3, *p* = 0.0038; DCXtr+, BrdU+/DCX+: both, *Z* = −3.068, *p* = 0.0038, Benjamini–Hochberg correction; Figure 6B]. LSt and CP differed in DCX+ cells, but not in BrdU+ cells, with higher levels of DCX+ cells in the LSt of pigeons [all MWU; DCXov+: *Z* = −3 *p* = 0.0038; DCXtr+, BrdU+/DCX+: both, *Z* = −3.068, *p* = 0.0038, Benjamini–Hochberg correction; Figure 6C]. If comparing GP, significant differences were found for DCX ov+ and DCX tr+ [all MWU; DCX ov+: *Z* = −3.013, *p* = 0.005; DCX tr.+: *Z* = −3072, *p* = 0.005; Benjamini–Hochberg correction; Figure 6D). Overall, the level of DCX+ cells was substantially lower in GP if compared to the striatal regions.

The analysis of adult striatal neuro‐ and gliogenesis, considering NeuN and GFAP in combination with BrdU as marker for adult‐born matured cells again showed profound differences between pigeons and mice (Figure 6E–H). Allover, higher levels of adult‐born neurons and glial cells were detected in striatal regions of pigeons. Comparison of ACB showed differences between BrdU+ and adult‐born glial cells marked with BrdU+/GFAP+ [all MWU, BrdU+, *Z* = −2.867, *p* = 0.006; BrdU+/GFAP+: *Z* = −3.068, *p* = 0.006, Benjamini–Hochberg correction; Figure 6E]. MSt and CP differed between all cell types, including adult‐born neurons and glia [all MWU, BrdU+: *Z* = −3, *p* = 0.0045; BrdU+/NeuN+: *Z* = −2.067, *p* = 0.039; BrdU+/GFAP+: *Z* = −3.068, *p* = 0.0045, Benjamini–Hochberg correction; Figure 6F]. BrdU+ and BrdU+/GFAP+ cells also differed between LSt and CP [all MWU, BrdU+: *Z* = −3, *p* = 0.0045; BrdU+/GFAP+: *Z* = −3.068, *p* = 0.0045, Benjamini–Hochberg correction; Figure 6G]. GP showed higher levels of BrdU+/NeuN+ cells in mice, while BrdU+/GFAP+ cells were higher in pigeons [MWU, BrdU+/NeuN+: *Z* = −2.879, *p* = 0.006; BrdU+/GFAP+: *Z* = −3.068, *p* = 0.006, Benjamini–Hochberg correction; Figure 6H].

### 3.3 Qualitative Analysis of the Proliferative Potential in the Subventricular Zone Proximal to the Caudate Nucleus of Macaques and Humans

A comprehensive study of the cytoarchitecture of the SVZ in both, the human and macaque brain, is provided to complement data in this understudied field and to underline the differences of the potential of human striatal adult neurogenesis compared to another primate species, the macaque. Thereby the analysis followed the delineation of the SVZ by Quinones‐Hinojosa et al. 2006, human) and Gil‐Perotin et al. 2009, macaque), but the SVZ and neighboring tissue of the caudate nucleus of both species was studied in more detail. The caudate nucleus is not only involved in several important motor and cognitive functions which may benefit from renewal or addable new neurons but it also develops differently in primate brains compared to rodents and birds (Kuenzel et al. 2011). This different development is accompanied in structural differences but also results in differences of “hot spots” of neurogenic niches in the SVZ. On the other hand, functionality of the caudate (and putamen) is still conserved across species.

Thus first, in order to obtain possible similarities and differences of the cellular structures in the SVZ of macaques and human, the biomarkers Sox2 (B‐/C‐cells), GFAP (B‐cells and glia) and Ki67 (B‐/C‐cells) were used that are relevant for initial neurogenesis steps (Niklison‐Chirou et al. 2020). In addition, the human caudate was further inspected with respect to other different neuronal stage markers Dlx‐2 (C‐/A‐cells), DCX (A‐cells and higher), NeuN and Calretinin (CR) for matured/integrated neurons as we could not observe any DCX+ signals in the macaque caudate nucleus.

#### 3.3.1 Macaque

**FIGURE 7:**
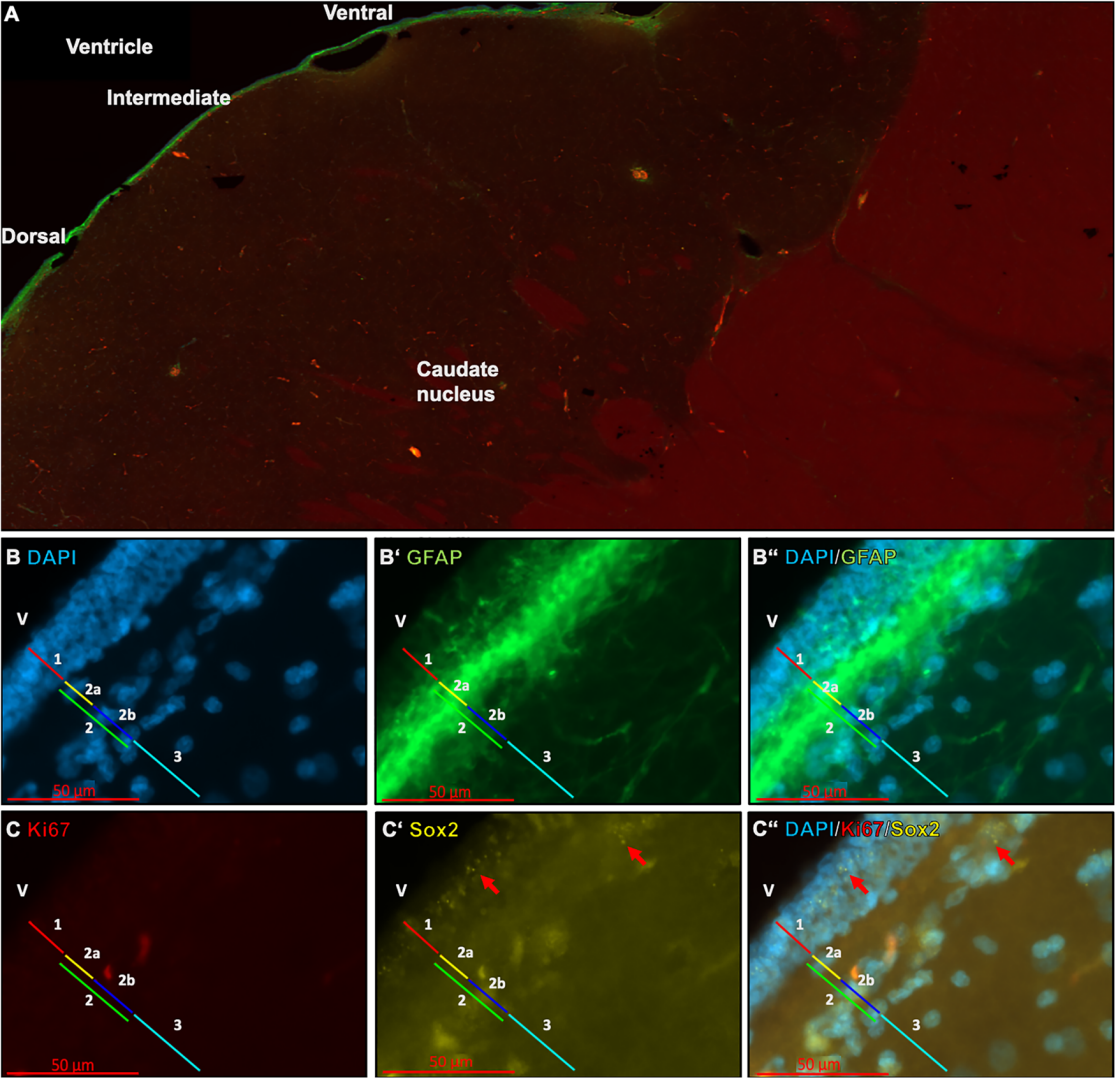
The subventricular zone of the caudate nucleus in the macaque. The ventral, intermediate, and dorsal parts of the SVZ are shown in close proximity to the neighboring ventricle and caudate nucleus (A). The section of the cutting plane shows the anterior horn of the lateral ventricle bordering the middle part of the lateral ventricle. B and C show the cellular architecture of the macaque SVZ on the lateral wall of the anterior–middle lateral ventricle, intermediate section with different cellular markers (GFAP/Ki67/Sox2/DAPI). 1: Ependymal layer; 2: GFAP+ dense layer; 2a: GFAP+ dense hypocellular layer with isolated cells; 2b: GFAP+ dense layer with band‐shaped GFAP cell bodies; 3: astrocytic band‐shaped layer. In C, red arrows indicate Sox2+ cells. V: Ventricle.

**FIGURE 8:**
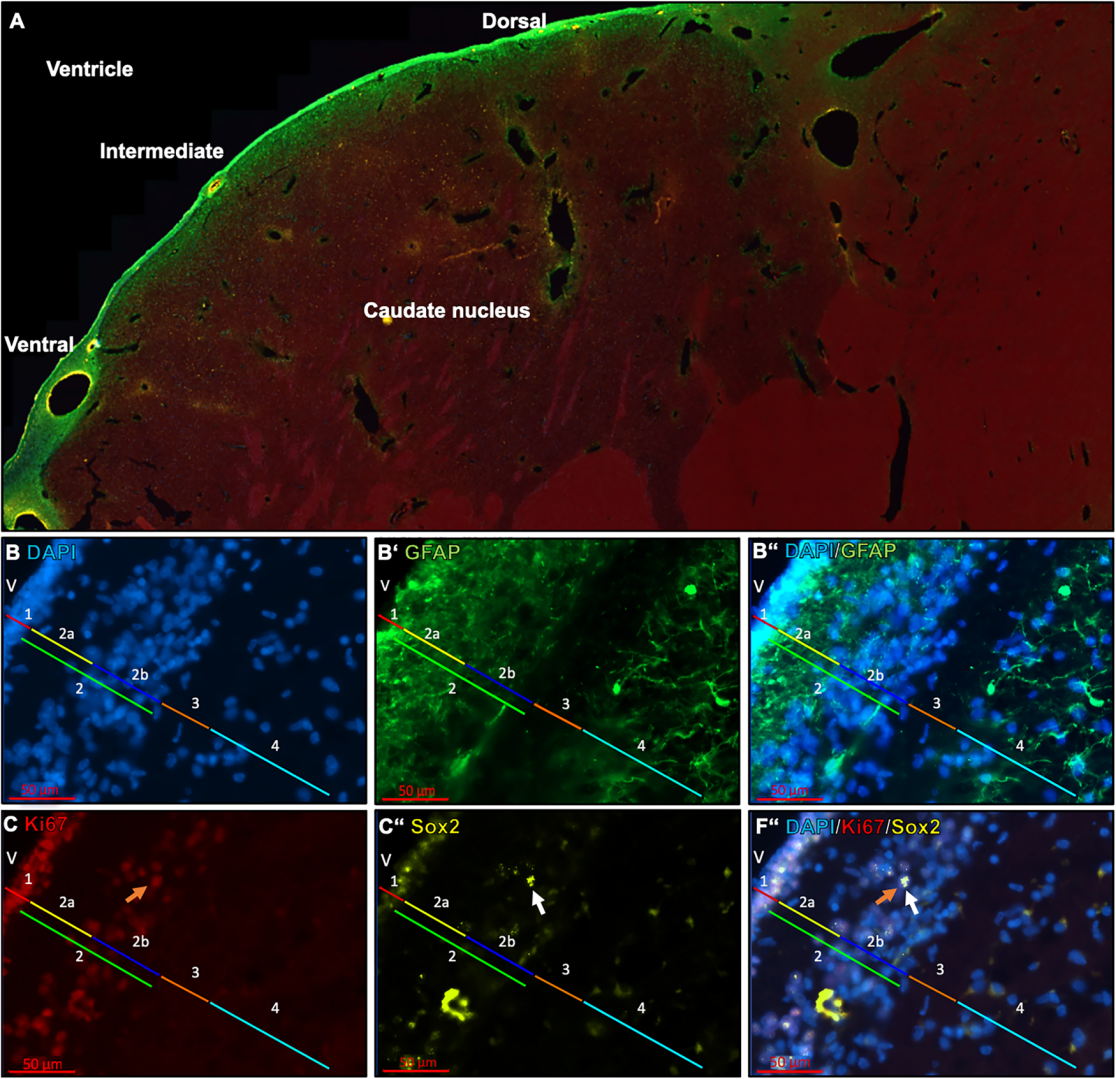
The subventricular zone of the caudate nucleus in the human. The ventral, intermediate, and dorsal parts of the SVZ are shown in close proximity to the neighboring ventricle (V) and caudate nucleus (A). The section of the cutting plane shows the anterior horn of the lateral ventricle bordering the middle part of the lateral ventricle. B and C show the cellular architecture of the human SVZ at the lateral wall of the anterior–middle lateral ventricle, intermediate section with different cellular markers (GFAP/Ki67/Sox2/DAPI). 1: Ependymal layer; 2: GFAP+ dense layer; 2a: GFAP+ dense, hypocellular layer with isolated cells, 2b: GFAP+ dense layer with band‐shaped GFAP cell bodies; 3: Discrete GFAP‐negative layer; 4: astrocytic band‐shaped layer. White arrows indicate cells that are Sox2+. Yellow arrows indicate cells that are GFAP+. Orange arrows indicate cells that are Ki67+.

A detailed overview of the SVZ of the macaque is provided in Figure 7A. The ependymal layer (1) was identified in the macaque preparations using the combination of DAPI and GFAP. This layer was neighbored by a GFAP+ rich layer (2) that was further subdivided into a hypocellular zone (2a) and a cell‐dense region (2b; Figure 7B,B’). In contrast to the human sample (Figure 8B), no discrete GFAP cell layer was recognized in the intermediate zone of the macaque sections. Instead, the astrocytic GFAP+ cell layer was immediately adjacent (Figure 7Bʼʼ). Ki67+ signaling was absent in the macaque samples (Figure 7C). However, isolated Sox2+ signals were detected in the ependymal cell layer as well as in the GFAP+ rich cell region (2b; Figure 7Cʼ,Cʼʼ). A variation in cellular layer thickness was also observed in the macaque samples, particularly at the transition to the dorsal and ventral sections of the SVZ. In none of our samples we detected DCX+ cells in the caudate nucleus of the macaques.

#### 3.3.2 Human

The study by Quinones‐Hinojosa et al. (2006) used a variety of biomarkers including DAPI, GFAP, DCX, NeuN, and Ki67. In addition, electron microscopic examinations were carried out to obtain detailed insights into the nanostructure of the SVZ. Here, we found similar results on the cytoarchitecture (microscale) of the SVZ of an individual human with some differences in cytoarchitecture and arrangement of the layers (Figure 8A). The ependymal layer (1) is clearly recognizable as the first layer (Figure 8B,C). The sections show that this layer is distinct and clearly demarcated and represents a boundary to the ventricle. This layer is neighbored by a layer that exhibits intense GFAP+ reactivity (2). The GFAP+ rich layer can be further subdivided into a hypocellular zone (2a) and a denser cellular layer (2b; Figure 8B¼,Bʼʼ). Both areas, the hypocellular zone and the denser cell region, are characterized by strong GFAP+ background signals. A discrete GFAP+ zone (3) can be recognized in the subsequent layer. Next, a loose cell cluster with prominent GFAP+ cell processes was identified (4), which becomes increasingly looser toward the brain parenchyma. The clear demarcation of these cell layers was detectable all over the intermediate regions of the SVZ. At the dorsal and ventral ends of the brain sections (Figure 8A), the ependymal, hypocellular and astrocytic layers in particular were continuously demarcated. However, the third, discrete GFAP+ zone is missing here. In addition, the thickness of these layers varies in the transition to the dorsal and ventral SVZ zone. In addition, Ki67+ signals were increasingly detectable in the ependymal layer (Figure 8C). Ki67+ signals were also observed sporadically in the dense cellular region of the GFAP+ rich layer (2b). Sox2 signals were pronounced in this layer, but present in smaller numbers (Figure 8C’). Some of these signals even showed triple staining with Ki67/Sox2/DAPI (Figure 8Cʼʼ).

**FIGURE 9:**
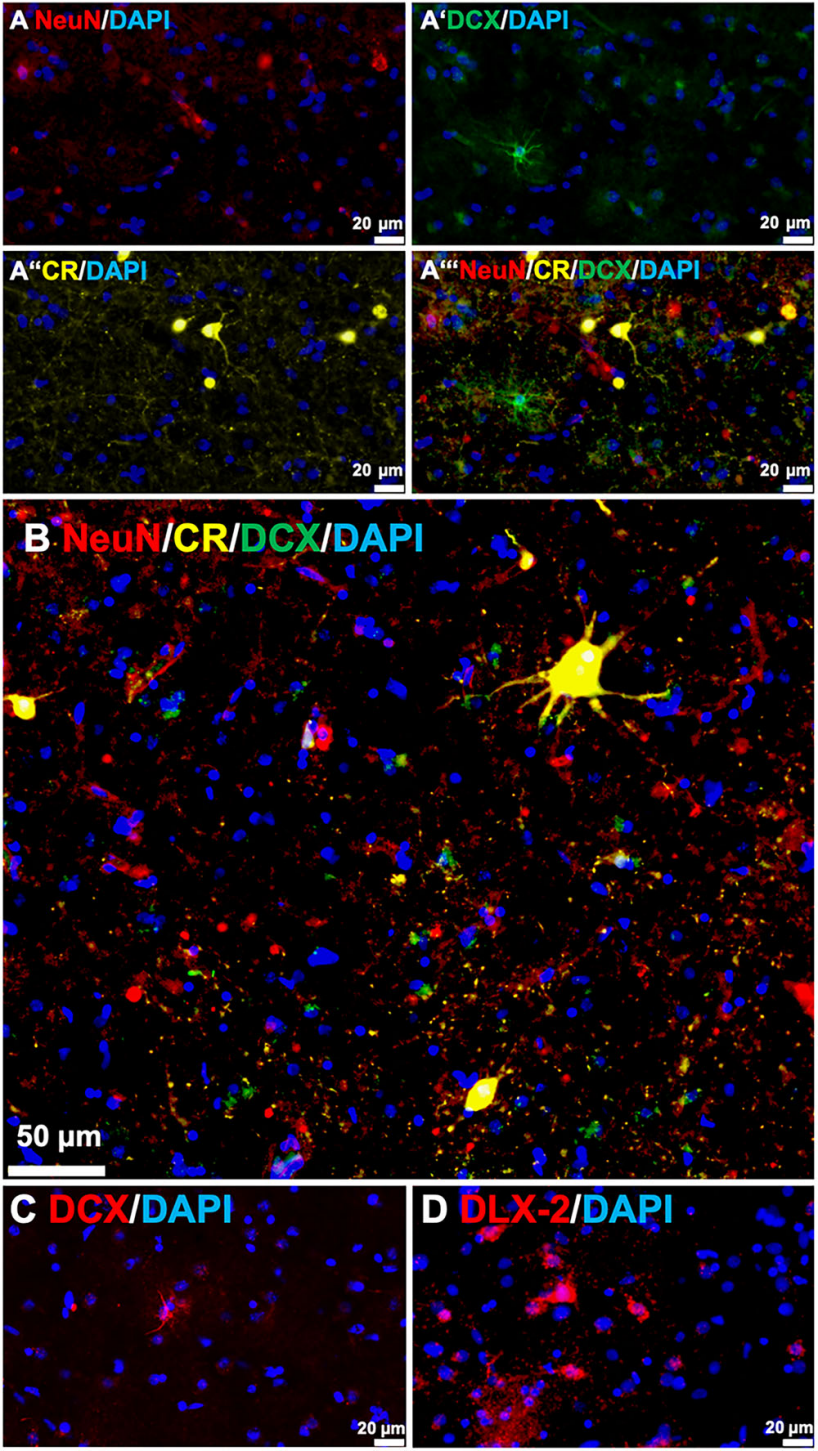
Signs for adult neurogenesis in the human caudate nucleus. Different markers were used to show different stages of not fully‐ and fully matured neurons. Triple labeling for NeuN (A, red), DCX (A’, green), Calretinin (CR, Aʼʼ, yellow), and combined marker illustration of the same detail (Aʼʼʼ). Aʼʼʼ additionally shows a double‐labeled NeuN/CR neuron (orange). At another position in the caudate nucleus (B), different sizes/types of CR+ neurons (yellow) are shown. Extensions of the largest CR+ neuron in the picture connect to DCX+ cells (green). C shows the ovoidal type of DCX+ cells (migrating). D shows an example of Dlx‐2 positive cells that are classified as stage 2/3 (C‐/A‐cells) during neuronal development from the neurogenic niche (s) in the SVZ. In all pictures, cell nuclei are further labeled with DAPI (blue).

To explore whether we find further stages of neurogenesis in the SVZ neighboring caudate or not, samples were stained against Dlx‐2, DCX, NeuN and CR to mark different stages of neuronal maturation and neuron types (Figure 9). Here, we focused on the anterior caudate located to the immediate vicinity of the SVZ. Cells that showed labeling for NeuN, mark fully matured neurons (Figure 9A), DCX marks neurons at either a proliferative or migrating state (Figure 9Aʼ,B,C) and CR signals classifies fully matured interneurons (Figure 9Aʼʼ). Some DCX+ cells show extensions that spread extensively out (Figure 9Aʼ), while others had only few extensions (migrating) or showed only cell body staining (Figure 9Aʼ,B). Many DCX+ radial fibers were additionally observed in the SVZ (not shown). CR+ signals were recognized in typically different sized striatal interneurons (Figure 9Aʼʼʼ,B). A few cells were observed that showed NeuN+/CR+ double labeling likely indicating another stage/neuron‐type (Figure 9Aʼʼʼ,B). Further, cases were observed where dendrites of large CR+ neurons seem to contact DCX+ cells in close neighborhood (Figure 9B). To additionally prove earlier neuronal developmental stages in the adult caudate, the stage 2/3 (C‐/A‐cells) marker Dlx‐2 was used. The detection of Dlx‐2+ cells confirmed that neurons in the adult caudate are still developing (Figure 9D).

## 4 Discussion

The results of this study revealed a different pattern of adult striatal neurogenesis in the individual subregions of pigeons and mice, while the observations in humans and macaques highlight the need to study specific questions in the future and again even showed differences in primates. The overall levels of striatal adult neurogenesis were significantly higher in pigeons than in mice, whereas in mice, even some of the investigated stages of neurogenesis studied were not detectable and others appeared to be very low. The same seems to be true for the macaques. However, to be fair, birds generally show high levels of adult neurogenesis in several brain regions (Barnea and Pravosudov 2011; Mazengenya et al. 2018; Herold et al. 2019; Mehlhorn et al. 2022; Brenowitz et al. 2024). On the other hand, now that we have quantified this differences in the striatum, at least for pigeons and mice, our results offer a new perspective to study the importance and functionality of striatal neurogenesis in the future. We would expect that in pigeons and likely other birds, effects based on environmental changes, hormones, stress, sex, cognition, pharmacological manipulation, or specific vector applications may become more prominent compared to rodents. Furthermore, as striatal newborn neurons have been detected at physiological levels in diverse species, studying this topic in birds may provide easier access to certain scientific questions, as studying primates becomes more difficult (Barnea and Pravosudov 2011; Ernst et al. 2014; Inta et al. 2015; Jurkowski et al. 2020; Elliott et al. 2025). Thus, even after a decade from the report of Ernst et al. (2014), less is known about the mechanisms that regulate striatal adult neurogenesis, how these newborn neurons integrate into long‐term established networks and whether they can modulate regular striatal functions. Only a few studies have reported that striatal adult neurogenesis is stimulated due to ischemia in rodents and macaques (Parent et al. 2002; Tonchev et al. 2005), while the fate of these neurons appears to be restricted to CR‐GABAergic interneurons (Yang et al. 2008; Liu et al. 2009). In addition, striatal adult neurogenesis appears to be depleted in Huntington's Disease, while depletion correlates to disease progression suggesting a role for preservation/protection in human (Ernst et al. 2014). Striatal adult neurogenesis may play a further role in psychiatric disorders shown in a mouse model (Inta et al. 2016), as well as in affective disorders associated with neuroinflammation, neurotrophic signaling and the microbiome in general (Alonso et al. 2024). Based on these reports and an increasing number of psychiatric disorders it seems to be a requisite to study underlying molecular and cellular mechanisms that link striatal adult neurogenesis to different pathological conditions into more detail, which will offer new fundamental insights into this process.

Based on the reported results, the ACB of pigeons exhibits the highest levels of striatal adult neurogenesis within the expression of DCX+, BrdU+/DCX+, and BrdU+/NeuN+. For example, DCX ovoidal cells are 64 times higher compared to mice. The finding of high numbers of DCX+ cells in the striatum of pigeons is in line with studies, which reported high densities of DCX+ cells and/or PCNA+ cells in the basal ganglia of pigeons without further specification of subdivisions (Melleu et al. 2013; Mazengenya et al. 2017). Here, the investigated ACB region is thereby considered part of the core region and belongs to the ventral striatum (Balint et al. 2011; Bruce et al. 2016). Species‐independent, the ACB is a key structure in the brain's reward system and acts as a functional interface between the limbic and motor systems (Mogenson et al. 1980; Veenman et al. 1995; Morrison et al. 2017). It further plays an essential role in various behaviors such as locomotion, learning, impulsivity, risk‐taking, feeding behavior, social interaction, sexual motivation as well as incentive and reward (Everitt et al. 1991; Swanson et al. 1997; Kuhnen and Knutson 2005; Kelley et al. 2005; Basar et al. 2010; Roberts et al. 2012; Hamel et al. 2017; Patterson et al. 2025). It significantly influences the choice of goal‐oriented actions and its core reinforces the perception of motivating stimuli, while the shell regions suppress actions that are considered irrelevant or unrewarding (Zaborszky et al. 1985; Heimer et al. 1991; Ambroggi et al. 2011; Kuenzel et al. 2011; Sicre et al. 2020). This optimizes efficiency in achieving goals. In addition, the ACB processes the results of actions, which in turn influence the direction for future actions (Floresco 2015). Against this background, the increased neuronal plasticity and the higher rate of adult neurogenesis in the ACB of pigeons compared to other subregions seem to be important for the continuous adaptation and optimization of goal‐directed actions. This may enable pigeons to respond efficiently to changing environmental conditions and reward stimuli. Thus, the ability to integrate new neurons into the ACB may support the modulation and fine‐tuning of behavioral strategies necessary to successfully adopt to complex environments. Interestingly, even mice exposed to chronic or inflammatory pain showed a higher number of newly formed DCX+ neuroblasts in the ACB compared to control animals (Garcia‐Gonzalez et al. 2021). The pathological changes in these mice increased migration and differentiation of neuroblasts, suggesting an adaptive response of the brain to chronic pain.

Dorsal striatal parts of the pigeon comprise the MSt and the LSt, which are considered as associative–limbic and sensorimotor striatal regions (Bruce et al. 2016). These subdivisions can be compared with the CP of the mouse that is functionally similarly integrated (Reiner et al. 2004b, 2004a; Bruce et al. 2016). Again, proliferation, plasticity, and maturation showed higher levels in pigeons compared to mice. While DCX ovoidal cells showed 64 times higher counts (in both, MSt and LSt), BrdU+/NeuN+ levels were doubled (in MSt) or even (in LSt) compared to the CP levels of mice. In contrast, no DCX triangular, BrdU+/DCX+ or BrdU+/GFAP+ cells were detected in mice, neither in the ACB nor the CP. In addition, total BrdU+ levels used as a marker for cell division, were almost four times lower in mice. Of course, the observed differences between mice and pigeons might be influenced by further conditions. It has been shown, at least in the hippocampus that adult neurogenesis underlies complex stimulation mechanisms based on different factors (Kempermann et al. 2004; Sherry and Hoshooley 2010; Ming and Song 2011; Kempermann et al. 2018; Augusto‐Oliveira et al. 2019; Armstrong et al. 2020). Further, our mice group was composed only of male mice, while the pigeon group included both sexes and thus, is more heterogeneous, which may have influenced the results. It has been reported that mice show sex differences in hippocampal adult neurogenesis (Yagi et al. 2020), while other reported no differences at this point (Tsao et al. 2023). Thus, including female mice could result in higher levels of persistent newly matured neurons, because male mice were reported to show only higher levels in initial steps of neurogenesis. In birds, sex differences are very species‐dependent and highly depend on captivity, seasonality, sociality, and accompanied functions like singing in song birds (Smulders 2002; Ball 2016; Guigueno et al. 2016; Diez et al. 2021; Rose et al. 2022). So, we might not be able to exclude that such sex differences played a role in the current study. Another important variable might be environmental factors, like higher or lower activity levels and stimulation due to housing conditions. While pigeons were allowed to fly freely around the loft and had more complex social interaction, male mice were kept in constant groups in environmental enriched cages (with play stuff and a spinning wheel that changed from week to week, but still retained) and in both, birds and rodents it is described that activity levels and constant changes in environments can influence adult neurogenesis (Schloesser et al. 2010; Pytte et al. 2012; Melleu et al. 2016; Brenowitz et al. 2024; Frechou et al. 2024). The housing conditions might have also influenced our observations in macaques and human regarding DCX or Ki‐67. While the inspected slices of human SVZ in close proximity to the caudate nucleus and the caudate itself showed several signals, macaques, which were kept long live under experimental conditions did not show any signals. However, a more detailed study in both primate species toward an analysis of all striatal areas along the anterior–posterior axis and higher sample numbers of human individuals should provide more information. On the other hand, with respect to environmental factors we would see this as an advantage for future studies in birds, as the possibility of using natural housing conditions like in the current study become closer to what is usually daily experienced by freely living humans. In relation to the ovoidal DCX+ cells, our results may further indicate that migration pathways show species‐specific adaptations along the studied striatal areas, which might depend on the position of the ACB/CP/MSt/LSt and/or the fate of these DCX+ neurons migrating to distant areas in different species. However, at the time this is only an assumption, which has to be studied into more detail in birds in the future. Something like the rostral‐migratory‐stream in rodents has yet not been described in birds, while in pigeons and canaries, intense neurogenic niches “hot spots” were seen at different positions along the anterior–posterior axis of the ventricular zones including different migrating paths at least in pallial areas and olfactory bulb (Alvarez‐Buylla et al. 1998; Melleu et al. 2013; Mehlhorn et al. 2022). However, to study this into detail might be a future endeavor requiring more data from individual animals as some striatal subregions like the ACB are relatively small (in pigeons and in mice) and analysis on that point with the current data set did not lead to congruent results in both species. For future prospective, particularly regarding ACB, it might be also necessary to include the possibility that neurogenic activity has been reported in the Islands of Calleja in mice (Saaltink et al. 2012) and birds might have similar or corresponding regions as reported in the developing chicken brain with Lmo4 expressing cell clusters (Abellan and Medina 2009).

In order to provide a first glimpse of a comparative view of important neurogenic niches in primates, the human and macaque SVZs analyzed in this article were subdivided into specific layers (Quinones‐Hinojosa et al. 2006; Gil‐Perotin et al. 2009). However, small differences in the exact categorization of the layers compared to the two studies cited above were identified. In accordance with Quinones‐Hinojosa et al. (2006) and Gil‐Perotin et al. (2009), the first layer of the SVZ is the ependymal layer. It is located directly at the lateral ventricles and represents the first boundary to the ventricle. In the previous studies, the ependymal layer is followed by a hypocellular layer, which was characterized by a high GFAP+ reactivity, followed by a third layer of astrocytes. In contrast, a zone with high GFAP+ reactivity was detected in the current study, which is subdivided: (i) into a hypocellular layer that contains only a few cells and (ii) a dense cell layer with GFAP cell bodies but also exhibited high GFAP+ background reactivity. Another special feature can be observed in the intermediate zone of the SVZ. Here, a third layer, the GFAP‐negative layer, can be detected between layers 2b and 4. This was referred to as a *gap layer* (Quinones‐Hinojosa et al. 2006). The exact identity of the cells present in layer 2b remains unclear. They could either be further misplaced ependymal cells or different types of glial cells. It is noteworthy that Ki67+ and Sox2+ signals are detectable in both, the ependymal layer and layer 2b. These markers could indicate the presence of proliferatively active progenitor cells, independent of their fate. The SVZ of macaques has similar characteristics. Although the layer thicknesses can be different, they are clearly demarcated from each other. In macaques, Sox2+ signals were found in the ependymal layer and layer 2b, which could indicate again the presence of progenitor cells. In contrast to the human brain slices, however, no Ki67+ signals were found in the SVZ of macaques. In comparison, it can be assumed that there are indeed cells in the ependymal layer and layer 2b of humans that are proliferatively active, which is either a sign of ongoing adult neurogenesis or oligodendrogenesis, suggesting that there is at least low proliferative activity in the SVZ of humans even in old age (Zhang et al. 2018). The hypocellular layer (2a) of human and macaque differs significantly from other species. The SVZ of the mouse, for example, is organized quite differently (Doetsch et al. 1997). As previously mentioned by Gil‐Perotin et al. (2009), the hypocellular region is considered an anatomical remnant that may have originally served as a migration pathway for newly formed neuroblasts to separate them from the rest of the brain parenchyma. To investigate this question in more detail, Sanai et al. (2011) conducted studies on the SVZ of humans of different ages and gained some significant insights. They reported that the human SVZ and the RMS represent a migration pathway for neuroblasts in the first 18 months of life. This activity decreases with increasing age and disappeared almost completely during adulthood. What remains is the hypocellular layer in the SVZ, which is detectable in adult individuals. In addition to the migration pathways for the olfactory bulb, a significant migration pathway to the prefrontal cortex was also identified (Sanai et al. 2011). This emphasizes the statement that the hypocellular zone in primates could be an early migration pathway that remains as a remnant in the adult brain. How this is developed in the bird brain should be an interesting question for future studies as birds show adult neurogenesis in several brain areas all over the telencephalon (Paton and Nottebohm 1984; Alvarez‐Buylla et al. 1990; Melleu et al. 2013; Robertson et al. 2017; Mazengenya et al. 2018; Herold et al. 2019; Mehlhorn et al. 2022;). As mentioned with respect to mice, studying the Islands of Calleja in primates including humans might be another interesting region to take into account (Meyer et al. 1989; Abellan and Medina 2009; Haber and Knutson 2010; Saaltink et al. 2012). In addition, even though it is only one human brain we studied, and we are aware of the role of intersubject variability, in particular in the human brain, it adds to the literature because as far as we have researched still only a few human brains were studied world‐wide regarding adult striatal neurogenesis (see Kempermann et al. 2018 or Alonso et al. 2024 for review). The brain from our study has undergone the same methodical procedures as applied for the other species studied and thus, we see it as necessary to include it for comparison.

Taken together the current study provides a comprehensive quantitative and qualitative analysis of adult neurogenesis in the striatum of pigeons and mice. The results suggest that birds could serve as a powerful model to study adult striatal neurogenesis in the future. In particular pigeons (but of course also other birds) are easy to handle and to hold, are very robust, outlive rodents many times over, have proved their comparability to certain neuroscientific issues regarding different functions, for example, learning, reward processing, navigation, moving/motor learning, visual perception and are very suitable for various methodological strategies, including viral vector applications, electrophysiological recordings, and calcium‐imaging (Scarf et al. 2011; Clark et al. 2022; Turner and Wassermann 2023; Wasserman et al. 2024; Güntürkün et al. 2024; Nimpf et al. 2024). Finally, increasing knowledge of different vertebrate species may help to shed some light into the discussion of the evolutionary traits of adult neurogenesis in general (Doetsch and Scharff 2001; Barnea and Pravosudov 2011; Bonfanti 2016; Kempermann et al. 2018). As we have partly replicated the potential for newly generated neurons in adult primates, as well as different stages of neurogenesis in the human caudate, we highly encourage to study this topic into more detail in the future. Further, with respect to the evolutionary aspects of adult neurogenesis, at a next step higher resolution microscopic analysis of the SVZ in different species may be useful to gain further insights that will improve the understanding of how adult striatal neurogenesis could be stimulated in the neurogenic niches to facilitate future studies.

## Author Contributions

Christina Herold wrote the manuscript and designed the experiments. Christina Herold, Nicole Delhaes, and Erhan Karsli established the immunohistochemical staining procedures. Erhan Karsli and Christina Herold analyzed the data and designed the figures. Hans Bidmon applied the cutting of brain blocks from macaque and human brains and provided advise for fixating and immunohistochemical staining of these blocks. Christina Herold and Julia Mehlhorn provided the pigeon brain series. Christina Herold, Erhan Karsli, Hans Bidmon, Katrin Amunts contributed to the interpretation of the data. All the authors read the manuscript, provided critical review, and contributed to the final version.

## Funding

This work was supported by a grant to Christina Herold from the research committee of the HHU, Germany (CH37/2010). The project has received further funding from the European Union's Horizon Europe Programme under the Specific Grant Agreement No. 101147319 (EBRAINS 2.0 Project; Katrin Amunts)

## Conflicts of Interest

The authors declare no conflicts of interest.

## Peer Review

The peer review history for this article is available at https://doi.org/10.1002/cne.70107.

## Supporting information




**Supporting Information Figure 1** Brdu+, DCXov+, DCXtri+ and BrdU+/DCX+ cells along the anterior–posterior axis of the pigeon brain. (A) ACB, (B) MSt, (C) LSt, (D) GP at the different atlas positions according to Karten and Hodos 1967. All numbers reflect the mean ± *SEM*. *p < 0.05 Wilcoxon signed‐rank test.

**Supporting Information Figure 2** Brdu+, BrdU+/NeuN+ and BrdU+/GFAP+ cells along the anterior–posterior axis of the pigeon brain. (A) ACB, (B) MSt, (C) LSt, (D) GP at the different atlas positions according to Karten and Hodos (1967). Data are presented as the mean ± *SEM*. *p < 0.05 Wilcoxon signed‐rank test.

**Supporting Information Table 1** Overview of immunohistochemical staining protocols

**Supporting Information Table 2** Distribution of BrdU+, DCXov+, DCXtri+, BrdU+/DCX+ cells/mm^2^ in the striatum of the pigeon. Values are mean values ± standard error.

**Supporting Information Table 3** Distribution of BrdU+, BrdU+/GFAP+, BrdU+/NeuN+ cells/mm^2^ in the striatum of the pigeon. Values are mean values ± standard error.

**Supporting Information Table 4** Distribution of BrdU+, DCXov+ cells/mm^2^ in the mouse striatum. Values are mean values ± standard error.

**Supporting Information Table 5** Distribution of BrdU+, BrdU+/GFAP+, BrdU+/NeuN+ cells/mm^2^ in the mouse striatum. Values are mean values ± standard error.

## Data Availability

The data that support the findings of this study are available in the main text, tables and figures.
