# Supplementary material for: Adult Striatal Neurogenesis—A Comparative Approach Between Pigeons, Mice, Macaques, and Human
Source: J Comp Neurol. 2025 Nov 2;533(11):e70107. doi: 10.1002/cne.70107 (PMC12580488; doi:10.1002/cne.70107)
Supplement: Supplementary file 2 — Supporting Information Figure 2 Brdu+, BrdU+/NeuN+ and BrdU+/GFAP+ cells along the anterior–posterior axis of the pigeon brain. (A) ACB, (B) MSt, (C) LSt, (D) GP at the different atlas positions according to Karten and Hodos (1967). Data are presented as the mean ± SEM. *p < 0.05 Wilcoxon signed‐rank test. [file CNE-533-e70107-s002.docx]

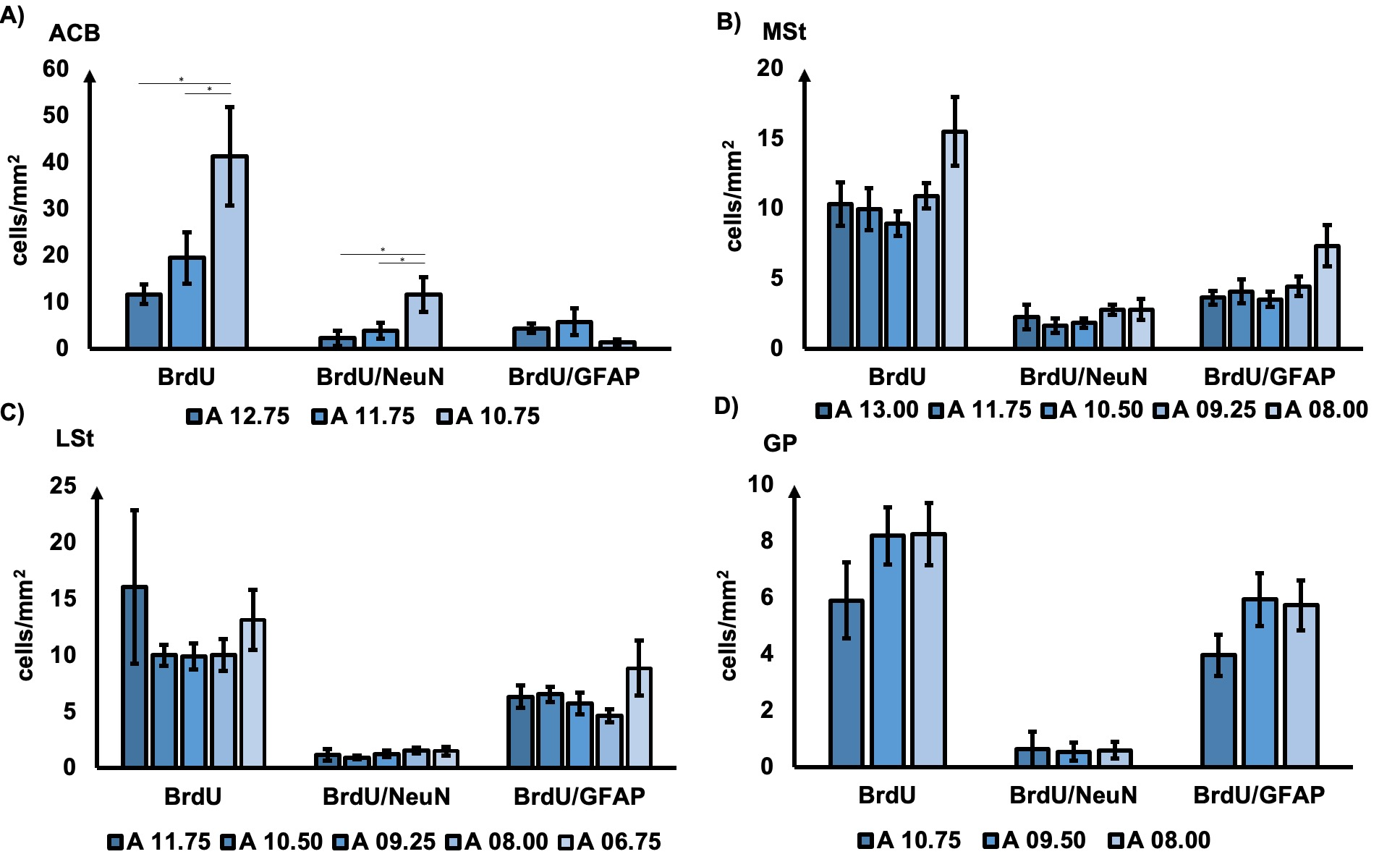


Suppl. Figure 2: Brdu+, BrdU+/NeuN+ and BrdU+/GFAP+ cells along the anterior-posterior axis of the pigeon brain. A) ACB, B) MSt, C) LSt, D) GP at the different atlas positions according to Karten and Hodos, 1967. Data is presented as the mean ± SEM. *p<0.05 Wilcoxon-rank test
