# Supplementary material for: Adult Striatal Neurogenesis—A Comparative Approach Between Pigeons, Mice, Macaques, and Human
Source: J Comp Neurol. 2025 Nov 2;533(11):e70107. doi: 10.1002/cne.70107 (PMC12580488; doi:10.1002/cne.70107)
Supplement: Supplementary file 3 — Supporting Information Table 1 Overview of immunohistochemical staining protocols [file CNE-533-e70107-s001.docx]

Suppl. Table 1 Overview of immunohistochemical staining protocols

|  | Human | Macaque | Pigeon | Mouse |
| --- | --- | --- | --- | --- |
| Preparation | Preparations in 0.12 M PBS | Preparations in 0.12 M PBS | Preparations in 0.12 M PBS | Preparations in 0.12 M PBS |
| Washing steps | 2 x 5 min. in 0.12 M PBS | 2 x 5 min. in 0.12 M PBS | 2 x 5 min. in 0.12 M PBS | 2 x 5 min. in 0.12 M PBS |
| DNA-Denaturation (only for BrdU-labeling) | - | - | 2N HCl, 45°C,  30 min. | 2N HCl, 45°C,  30 min. |
| pH increase | - | - | 10 min. in 0.1 M borate-buffer (pH 8.5) | 10 min in. 0.1 M borate-buffer (pH 8.5) |
| Washing steps | - | - | 2 x 10 min. in  0.12 M PBS | 1 x 10 min. in  0.12 M PBS |
| Blocking | 60 min. in PBS-T with 10%  Goat/donkey serum | 60 min. in PBS-T with 10%  Goat/donkey serum | 60 min. in PBS-T with 10% goat serum | 60 min. in PBS-T with 10% goat serum |
| Primary- antibody-Mix | overnight at 4°C | overnight at 4°C | overnight at 4°C | overnight at 4°C |
| Washing steps | 3 x 10 min.  in 0.12 M PBS | 3 x 10 min.  in 0.12 M PBS | 3 x 10 min.  in 0.12 M PBS | 3 x10 min.  in 0.12 M PBS |
| Secondary- antibody | 2 h RT | 2 h RT | 2 h RT | 2 h RT |
| Washing steps | 3 x 10 min.  in 0.12 M PBS | 3 x 10 min.  in 0.12 M PBS | 3 x 10 min.  in 0.12 M PBS | 3 x 10 min. in 0.12 M PBS |
| Lipofuscin Eliminator | Autofluorescence Eliminator-Reagent | Autofluorescence Eliminator-Reagent |  |  |
| Core colouring | DAPI | DAPI | - | -/Hoechst (1:2000),  30 min. RT |
| Washing steps | 3 x 10 min. in PBS  2 x 5 min in PB | 3 x 10 min. in PBS  2 x 5 min in PB | 3 x 5 min in PBS  2x 5 min in PB | 3 x 10 min. in PBS  2 x 5 min in PB |
| Covering | Mounting Solution, dry in the dark, DAPI | Mounting Solution, dry in the dark, DAPI | Mounting Solution, dry in the dark, Fluoromount | Mounting Solution, dry in the dark, Fluoromount |
