# Supplementary material for: Adult Striatal Neurogenesis—A Comparative Approach Between Pigeons, Mice, Macaques, and Human
Source: J Comp Neurol. 2025 Nov 2;533(11):e70107. doi: 10.1002/cne.70107 (PMC12580488; doi:10.1002/cne.70107)
Supplement: Supplementary file 4 — Supporting Information Table 2 Distribution of BrdU+, DCXov+, DCXtri+, BrdU+/DCX+ cells/mm2 in the striatum of the pigeon. Values are mean values ± standard error. [file CNE-533-e70107-s007.docx]

Suppl. Table 2: Distribution of BrdU+, DCXov+, DCXtri+, BrdU+/DCX+ cells/mm^2^ in the striatum of the pigeon. Values are mean values +/- standard error.

| **Striatal region** | **BrdU+** | **DCXov+** | **DCXtri+** | **BrdU+/DCX+** |
| --- | --- | --- | --- | --- |
| **ACB** | 17,81 ± 2,84 | 255,04 ± 17,53 | 8,39 ± 1,48 | 1,65 ± 0,21 |
| **MSt** | 4,75 ± 0,29 | 161,04 ± 9,33 | 13,19 ± 0,55 | 0,73 ± 0,09 |
| **LSt** | 3,10 ± 0,25 | 103,49 ± 5,98 | 11,37 ± 0,59 | 0,46 ± 0,04 |
| **ISt** | 4,12 ± 0,99 | 18,83 ± 2,12 | 1,12 ± 0,44 | 0,18 ± 0,12 |
| **GP** | 2,56 ± 0,29 | 17,10 ± 1,36 | 1,28 ± 0,16 | 0,16 ± 0,06 |
