# Supplementary material for: Adult Striatal Neurogenesis—A Comparative Approach Between Pigeons, Mice, Macaques, and Human
Source: J Comp Neurol. 2025 Nov 2;533(11):e70107. doi: 10.1002/cne.70107 (PMC12580488; doi:10.1002/cne.70107)
Supplement: Supplementary file 5 — Supporting Information Table 3 Distribution of BrdU+, BrdU+/GFAP+, BrdU+/NeuN+ cells/mm2 in the striatum of the pigeon. Values are mean values ± standard error. [file CNE-533-e70107-s004.docx]

Suppl. Table 3: Distribution of BrdU+, BrdU+/GFAP+, BrdU+/NeuN+ cells/mm^2^ in the striatum of the pigeon. Values are mean values +/- standard error.

| **Striatal region** | **BrdU+** | **BrdU+/GFAP** | **BrdU+/NeuN+** |
| --- | --- | --- | --- |
| **ACB** | 23,17 ± 4,9 | 3,42 ± 0,59 | 5,38 ± 1,85 |
| **MSt** | 10,37 ± 1,01 | 4,11 ± 0,39 | 2,22 ± 0,39 |
| **LSt** | 10,34 ± 1,16 | 6,1 ± 0,59 | 1,29 ± 0,22 |
| **ISt** | 8,93 ± 1,63 | 7,8 ± 1,59 | 0,23 ± 0,15 |
| **GP** | 7,79 ± 0,95 | 5,41 ± 0,68 | 0,56 ± 0,25 |
