# Supplementary material for: Adult Striatal Neurogenesis—A Comparative Approach Between Pigeons, Mice, Macaques, and Human
Source: J Comp Neurol. 2025 Nov 2;533(11):e70107. doi: 10.1002/cne.70107 (PMC12580488; doi:10.1002/cne.70107)
Supplement: Supplementary file 6 — Supporting Information Table 4 Distribution of BrdU+, DCXov+ cells/mm2 in the mouse striatum. Values are mean values ± standard error. [file CNE-533-e70107-s003.docx]

**Suppl. Table 4:** *Distribution of BrdU+, DCXov+ cells/mm^2^ in the mouse striatum.* Values are mean values +/- standard error.

| **Striatal regions** | **BrdU+** | **DCXov+** |
| --- | --- | --- |
| **ACB** | 2,77 ± 0,62 | 2,59 ± 0,45 |
| **CP** | 3,07 ± 0,57 | 1,62 ± 0,13 |
| **FS** | 7,99 ± 5,23 | 1,15 ± 0,79 |
| **GP** | 6,54 ± 2,15 | 0,14 ± 0,09 |
