# Supplementary material for: Adult Striatal Neurogenesis—A Comparative Approach Between Pigeons, Mice, Macaques, and Human
Source: J Comp Neurol. 2025 Nov 2;533(11):e70107. doi: 10.1002/cne.70107 (PMC12580488; doi:10.1002/cne.70107)
Supplement: Supplementary file 7 — Supporting Information Table 5 Distribution of BrdU+, BrdU+/GFAP+, BrdU+/NeuN+ cells/mm2 in the mouse striatum. Values are mean values ± standard error. [file CNE-533-e70107-s006.docx]

Suppl.Table 5: Distribution of BrdU+, BrdU+/GFAP+, BrdU+/NeuN+ cells/mm^2^ in the mouse striatum. Values are mean values +/- standard error.

| **Striatal regions** | **BrdU+** | **BrdU/NeuN** |
| --- | --- | --- |
| **ACB** | 5,23 ± 0,61 | 2,06 ± 0,56 |
| **CP** | 2,78 ± 0,32 | 1,04 ± 0,19 |
| **FS** | 7,37 ± 1,96 | 2,45 ± 0,77 |
| **GP** | 7,57 ± 0,56 | 3,28 ± 0,76 |
